# Gut microbial degradation of organophosphate insecticides-induces glucose intolerance *via* gluconeogenesis

**DOI:** 10.1186/s13059-016-1134-6

**Published:** 2017-01-24

**Authors:** Ganesan Velmurugan, Tharmarajan Ramprasath, Krishnan Swaminathan, Gilles Mithieux, Jeyaprakash Rajendhran, Mani Dhivakar, Ayothi Parthasarathy, D.D. Venkatesh Babu, Leishman John Thumburaj, Allen J. Freddy, Vasudevan Dinakaran, Shanavas Syed Mohamed Puhari, Balakrishnan Rekha, Yacob Jenifer Christy, Sivakumar Anusha, Ganesan Divya, Kannan Suganya, Boominathan Meganathan, Narayanan Kalyanaraman, Varadaraj Vasudevan, Raju Kamaraj, Maruthan Karthik, Balakrishnan Jeyakumar, Albert Abhishek, Eldho Paul, Muthuirulan Pushpanathan, Rajamani Koushick Rajmohan, Kumaravel Velayutham, Alexander R. Lyon, Subbiah Ramasamy

**Affiliations:** 1 Department of Molecular Biology, Centre for Excellence in Genomic Sciences, School of Biological Sciences, Madurai Kamaraj University, Madurai, Tamil Nadu 625021 India; 2 Center for Molecular and Translational Medicine, Research Science Center, Georgia State University, Atlanta, GA 30303 USA; 3 KMCH Research Foundation, Kovai Medical Centre and Hospital, Coimbatore, Tamil Nadu 641014 India; 4 Institut National de la Santé et de la Recherche Médicale, U1213, Lyon, 69372 France; 5 Department of Genetics, Centre for Excellence in Genomic Sciences, School of Biological Sciences, Madurai Kamaraj University, Madurai, Tamil Nadu 625021 India; 6 Department of Immunology, Centre for Excellence in Genomic Sciences, School of Biological Sciences, Madurai Kamaraj University, Madurai, Tamil Nadu 625021 India; 7 Deparment of Zoology, Madras Christian College, Chennai, Tamil Nadu 600059 India; 8 Department of Biochemistry, Centre for Excellence in Genomic Sciences, School of Biological Sciences, Madurai Kamaraj University, Madurai, Tamil Nadu 625021 India; 9 Department of Microbial Technology, Centre for Excellence in Genomic Sciences, School of Biological Sciences, Madurai Kamaraj University, Madurai, Tamil Nadu 625021 India; 10 Laboratory of Gene Regulation and Development, Program in Cellular Regulation and Development, National Institute of Child Health and Human Development, NIH, Bethesda, MD 20892 USA; 11 Institute of Diabetes & Endocrinology, Alpha Hospital and Research Centre, Madurai, Tamil Nadu 625009 India; 12 NIHR Cardiovascular Biomedical Research Unit, Royal Brompton Hospital and Imperial College, London, UK

**Keywords:** Organophosphates, Gut microbiota, Diabetes, Glucose intolerance, Acetic acid, Gluconeogenesis, Fecal transplantation, Metatranscriptomics, Metabolomics

## Abstract

**Background:**

Organophosphates are the most frequently and largely applied insecticide in the world due to their biodegradable nature. Gut microbes were shown to degrade organophosphates and cause intestinal dysfunction. The diabetogenic nature of organophosphates was recently reported but the underlying molecular mechanism is unclear. We aimed to understand the role of gut microbiota in organophosphate-induced hyperglycemia and to unravel the molecular mechanism behind this process.

**Results:**

Here we demonstrate a high prevalence of diabetes among people directly exposed to organophosphates in rural India (n = 3080). Correlation and linear regression analysis reveal a strong association between plasma organophosphate residues and HbA1c but no association with acetylcholine esterase was noticed. Chronic treatment of mice with organophosphate for 180 days confirms the induction of glucose intolerance with no significant change in acetylcholine esterase. Further fecal transplantation and culture transplantation experiments confirm the involvement of gut microbiota in organophosphate-induced glucose intolerance. Intestinal metatranscriptomic and host metabolomic analyses reveal that gut microbial organophosphate degradation produces short chain fatty acids like acetic acid, which induces gluconeogenesis and thereby accounts for glucose intolerance. Plasma organophosphate residues are positively correlated with fecal esterase activity and acetate level of human diabetes.

**Conclusion:**

Collectively, our results implicate gluconeogenesis as the key mechanism behind organophosphate-induced hyperglycemia, mediated by the organophosphate-degrading potential of gut microbiota. This study reveals the gut microbiome-mediated diabetogenic nature of organophosphates and hence that the usage of these insecticides should be reconsidered.

**Electronic supplementary material:**

The online version of this article (doi:10.1186/s13059-016-1134-6) contains supplementary material, which is available to authorized users.

## Background

Organophosphates (OPs) are esters, amides, or thiol derivatives of phosphoric acid synthesized first in the early 19th century. The history of the development of OPs is amalgamated with wars [1]. They are used as pesticides in agricultural fields, as chemical weapons in war fields, as plasticizers, oil additives, and lubricants in industries. Due to the advent of “Silent Spring” [2] and other environmental movements, organochlorine pesticides like DDT were banned and that place was strongly grasped by OPs in the 1970s [3]. Eventually, OP has become a largely used insecticide in the world, accounting for more than 40% of the pesticide market. Often less than 0.1% of pesticides sprayed are estimated to reach the target organism [4], while the remainder is deposited on plant surfaces or tissues, soil, water, and air and ultimately reaches off-target organisms including humans. The existence of OP residues in different media including air, soil, water bodies, vegetables, blood, urine, and tissues of humans and other animals were detected worldwide [5, 6].

In 1962, Carson designated OPs as one of the most poisonous chemicals of the world [2]. OPs inhibit acetylcholine esterase (AChE) [7], which in turn induces synapses of nervous and muscular systems leading to agitation, hypersalivation, convulsion, respiratory failure, and eventually death of insects and mammals. However, various animal [8–10] and human studies [11–14] have uncovered the association between OP exposure and diabetic prevalence. OP-metabolizing microbes have been identified in soil and other environments [15] and intestinal bacteria were proven to degrade OP [16]. Exposure to chronic OPs induces gut microbial dysbiosis [17] and intestinal dysfunctions [18]. Trillions of microbes constituting the gut microbiota represent a vast and rare repository of diet [19, 20] and xenobiotics metabolizing machinery [21, 22]. Alterations in ecology and physiology of gut microbiota affects the host metabolism and thereby determines the transition between health and disease [23] including diabetes [24, 25]. For instance, gut microbiota was proven to mediate the glucose intolerance induced by non-caloric artificial sweeteners [26]. Thus, the present study is designed to explore the effect of chronic OP exposure on glucose homeostasis and to identify the role of gut microbiota in OP-induced hyperglycemia.

## Results

### Plasma OP residues associated with human diabetes

**Fig. 1:**
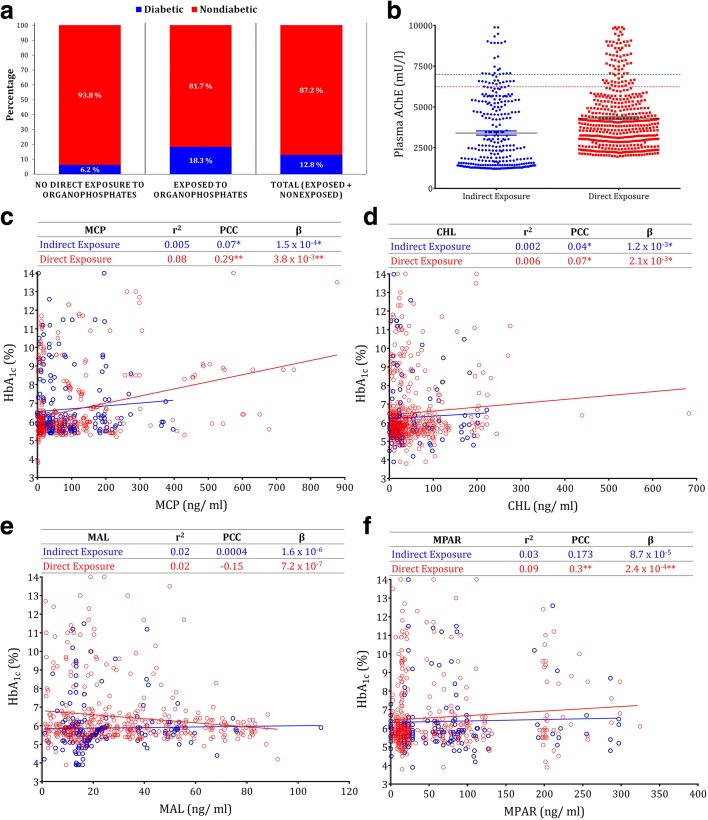
Blood plasma OP residues correlate with self-reported exposure and diabetic status. **a** Prevalence of diabetics among humans exposed to OP (n *=*1686) and not directly exposed to OP (n = 1394). The percentage of diabetic prevalence and non-prevalence are mentioned in the *bars*. **b** Plasma acetylcholine esterase (AChE) of people indirectly exposed (n = 303) and directly exposed (n = 499) to OP. *Dotted lines* represent the reference values for males (*green*) and females (*pink*). Regression plot of plasma OP residues vs. blood HbA_1c_ indirectly exposed (n = 303) and directly exposed (n = 499) to (**c**) MCP, (**d**) CHL, (**e**) MAL, (**f**) MPAR. *Horizontal lines* represent mean; *error bars* represent s.e.m; *P < 0.05 Rank sum, Mann–Whitney U Test (**b**). The *hollow* *﻿﻿circle﻿﻿ *represents individual values and *straight line* represents the trend line. *P < 0.05; **P < 0.01. *PCC* Pearson correlation coefficient, *β* regression coefficient (**c**–**f**)

Based on the survey executed among the pesticide users including pesticide applicators, farmers, and pesticide sellers in the villages of Vadapalanji Panchayat (Additional file 1: Figure S1), we found OP constitutes nearly 50% of insecticide usage (Additional file 1: Figure S2A, Additional file 2: Table S1). Among the OPs, monocrotophos (MCP), chlorpyrifos (CHL), malathion (MAL), and methyl parathion (MPAR) are frequently and extensively used (Additional file 2: Table S1). We executed another survey among the people (n = 3080) in the same villages. Age, sex, self-reported diabetic status, familial diabetic, and OP exposure history were collected from this rural population (Additional file 2: Table S2). Our survey indicates the high prevalence of diabetics (18.3%) among the people directly exposed to OP insecticides while it was threefold lesser (6.2%) among the indirectly exposed group (Fig. 1a, Additional file 1: Figure S1B; adjusted odds ratio (OR), 1.4; 95% confidence interval (CI), –0.74 to 2.47; Additional file 2: Table S3). To study the influence of genetic factors, we analyzed the familial diabetic history and found half of the diabetics in both groups had no familial diabetic history (Additional file 1: Figure S1C).

To validate the OP exposure and self-reported diabetic status, blood samples were collected from a random subpopulation (n = 802) that included non-diabetic (n = 554) and diabetic (n = 228) individuals. Details of age, sex, height, weight, pesticide exposure history, diabetic history, smoking, alcohol and tobacco usage, medications, and other disorders were collected using a standard questionnaire (Additional files 2: Table S4 and Additional file 3). The diabetic status was validated by HbA_1c_ analysis and people with HbA_1c_ ≥ 6.5 were considered as diabetic. Ninety percent of the self-reported diabetic status coincided with HbA_1c_ level (Additional file 2: Table S4), the rest of them were under hypoglycemic medications and hence considered as diabetic. In addition, 19% of the self-reported non-diabetic participants in an earlier study were newly diagnosed as diabetic in HbA_1c_ analysis that indicates the higher burden of diabetes in this rural community. To further validate the OP exposure, we studied plasma AChE, which is the target of OPs. Though 14.7% of the population had AChE above reference limits, they were distributed in both direct exposure and indirect exposure groups (6:4 ratio) with no significant association (Rank sum *P <* 0.09, Fig. 1b). Similarly, no significant association was observed in plasma AChE between diabetic and non-diabetic individuals (Rank sum *P <* 0.40, Additional file 1: Figure S3A).

The OP residues in the blood plasma were studied by GC/MS and m/z fragments specific to each OP are monitored by a single ion mode (Additional file 1: Figure S4). MCP, CHL, MAL, and MPAR residues were detected in 87.3%, 73.2%, 70.9%, and 68.3% of the study population, respectively. Nearly 70% of the samples with OP residues below detectable limits falls under the indirectly exposed category (Additional file 2: Table S4). No significant correlation was found between the total OP residues and plasma AChE level (Pearson Correlation co-efficient (PCC) = 0.04, *P* = 0.22) and BMI (PCC = −0.06, *P* = 0.07) (Additional file 2: Table S5).

On the other hand, after adjustment for confounding variables, significant positive correlation was observed between plasma OP residues (except MAL) and HbA_1c_ (Fig. 1c–e, Additional file 1: Figure S3B). The correlation was stronger in the case of the direct exposure group (PCC = 0.29, *P* < 0.01 for MCP; PCC = 0.07, *P* < 0.05 for CHL; PCC = −0.15, *P* = 0.08 for MAL; PCC = 0.3, *P* < 0.01 for MPAR) than the indirect exposure group (PCC = 0.07, *P* < 0.05 for MCP; PCC = 0.04, *P* < 0.05 for CHL; PCC = 0.0004, *P* = 0.06 for MAL; and PCC = 0.173, *P* = 0.07 for MPAR) (Fig. 1c–e). For every unit increase in the level of plasma OP residues, a corresponding increase in HbA_1c_ value was found by linear regression analysis. The regression co-efficients (β) for direct exposure category were 3.8 × 10^−3^ (*P* < 0.01), 2.1 × 10^−3^ (*P* < 0.05), 7.2 × 10^−7^ (*P* = 0.08), and 2.4 × 10^−4^ (*P* < 0.01) for MCP, CHL, MAL, and MAPR, respectively. In the case of the indirect exposure category, the β values were 1.5 × 10^−4^ (*P* < 0.05), 1.2 × 10^−3^ (*P* < 0.05), 7.2 × 10^−7^ (*P* = 0.06), and 8.7 × 10^−5^ (*P* = 0.07) for MCP, CHL, MAL, and MAPR, respectively (Fig. 1c–e).

By logistic regression, the multivariable adjusted ORs for diabetes associated with the highest quartile of each OP compared with its lowest quartile were 1.70 (95% CI, 0.86–1.37) for MCP (P_trend_ < 0.001), 1.82 (0.31–1.25) for CHL (P_trend_ < 0.05), 1.08 (0.54–2.16) for MAL (P_trend_ = 0.654), and 2.67 (1.23–2.80) for MPAR (P_trend_ < 0.05) (Additional file 2: Table S6). People with OP residues in the highest quartile were largely diabetic in the case of all four OPs (Additional file 1: Figure S5, Additional file 2: Table S7). Similarly, more than 50% of people in the highest quartile of OPs were from the directly exposed group and OP residues below the detectable limit were largely distributed among the group with no direct exposure to OPs (Additional file 1: Figure S6, Additional file 2: Table S7). Altogether, this study indicates a probable association between OP accumulation and diabetic prevalence with no change in AChE activity.

### Chronic OP impairs glucose tolerance and induces oxidative stress

Since the human studies indicate the association between plasma OP residues and diabetes prevalence, we examined whether chronic exposure to OP is a risk factor for hyperglycemia by studying a preclinical model of OP exposure in *BALB/c* mice. MCP is identified as the most frequently used insecticide in our survey (Additional file 2: Table S1) and as our previous study indicates that this OP induces hyperglycemia [10], we employed MCP as the prototypical OP. Mice were administered MCP orally at 10× theoretical maximum daily intake (TMDI) dose (28 μg/kg body weight/day) directly in drinking water (Additional file 1: Figure S7A). TMDI for MCP (0.17 mg/day) [6] was calculated as per WHO recommendations (1997) based on the maximum residue limits (MRL) available for selected grains and vegetables. Since, the level of residues in water, air, and other eatables are not considered for TMDI calculation, we provided 10X TMDI dose. The animals were provided pure water or MCP mixed water continuously for 180 days, which is equivalent to 12–15 years of human life.

**Fig. 2:**
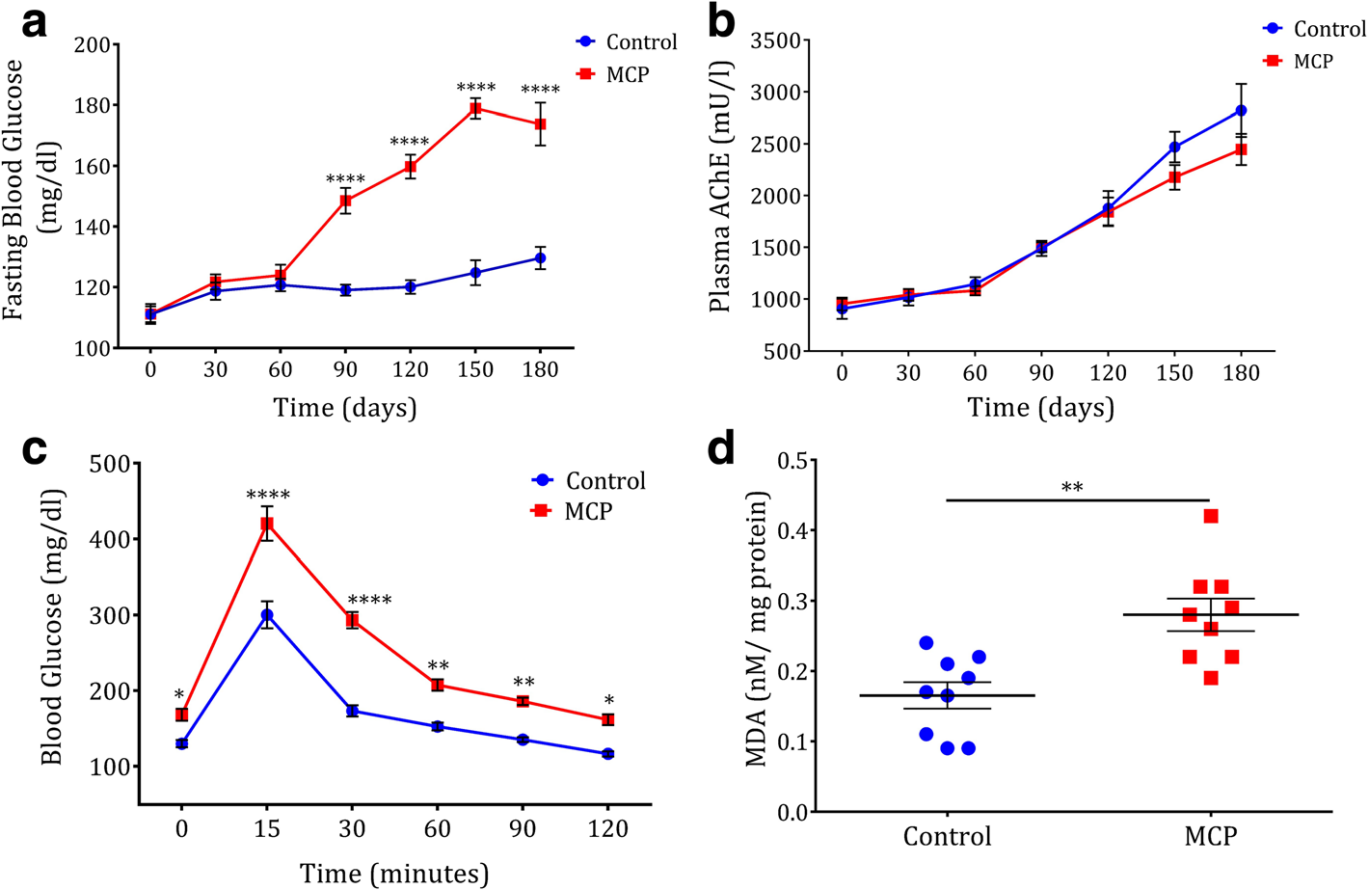
Chronic intake of OP-induces hyperglycemia and glucose intolerance leading to oxidative stress **a** Periodical fasting blood glucose of animals drinking pure water or MCP mixed water (n = 09). **b** Periodical plasma AChE level of animals drinking pure water or MCP mixed water (n = 10). **c** Oral glucose tolerance test (OGTT) of animals drinking pure water or MCP mixed water after 180 days (n = 09). **d** Serum lipid peroxidation level of animals after 180 days drinking pure water or MCP mixed water (n = 09). *Horizontal lines* or *symbols* represent mean; *error bars* represent s.e.m; ****P < 0.0001, **P < 0.01, P < 0.05. Unpaired two-sided student-t test. Experiments were repeated twice/thrice

No significant variation in body weight (*P <* 0.9999; Additional file 1: Figure S7B), food, and water intake (data not shown) were noticed between the control and MCP-fed animals. MCP-fed animals exhibited slow and steady increase in blood glucose levels, especially after 60 days (*P <* 0.0001; Fig. 2a), and exhibited significant hyperglycemia after 180 days (*P <* 0.0001; Fig. 2a, Additional file 1: Figure S7C, and Additional file 4: Table S10). However, no change was observed in the circulating level of AChE activity (*P <* 0.9999; Fig. 2b, Additional file 1: Figure S7D, and Additional file 4: Table S11). MCP-fed animals also showed impaired glucose tolerance compared to untreated controls (*P <* 0.0001; Fig. 2c, Additional file 1: Figure S7E, and Additional file 4: Table S12). The experiments were repeated thrice/twice and no significant variation between the batches was noticed (Additional file 4: Table S10–S12). We observed similar levels of fasting insulin between the two groups of animals (*P <* 0.50; Additional file 1: Figure S7F).

Hyperglycemia induces oxidative stress and previous studies reported the association between OP exposure and oxidative stress [8, 10, 27]. We noticed a significant elevation in lipid peroxidation (*P <* 0.002; Fig. 2d) and protein carbonylation (*P <* 0.02; Additional file 1: Figure S8A) in the serum of MCP-fed animals. A significant increase of lipid peroxidation in the liver (*P <* 0.003) and kidneys (*P <* 0.04; Additional file 1: Figure S8B) and increased protein carbonylation in liver of MCP-fed animals were observed (*P <* 0.02; Additional file 1: Figure S8C). In addition, increased total antioxidant activity was detected in the serum (*P <* 0.05; Additional file 1: Figure S3D) of MCP-fed animals. Increased levels of hepatic damage markers (LDH (*P <* 0.03), AST (*P <* 0.05), and ALT (*P <* 0.04)) in serum (Additional file 1: Figure S8E) suggests free radicals mediated tissue damage. Histopathology of the liver showed signs of hepatic periportal inflammation and fibrosis (Additional file 1: Figure S8F). Collectively, these results indicate that chronic OP exposure induces glucose intolerance leading to oxidative stress and hepatic damage.

### OP-induced glucose intolerance mediated by gut microbiome

**Fig. 3:**
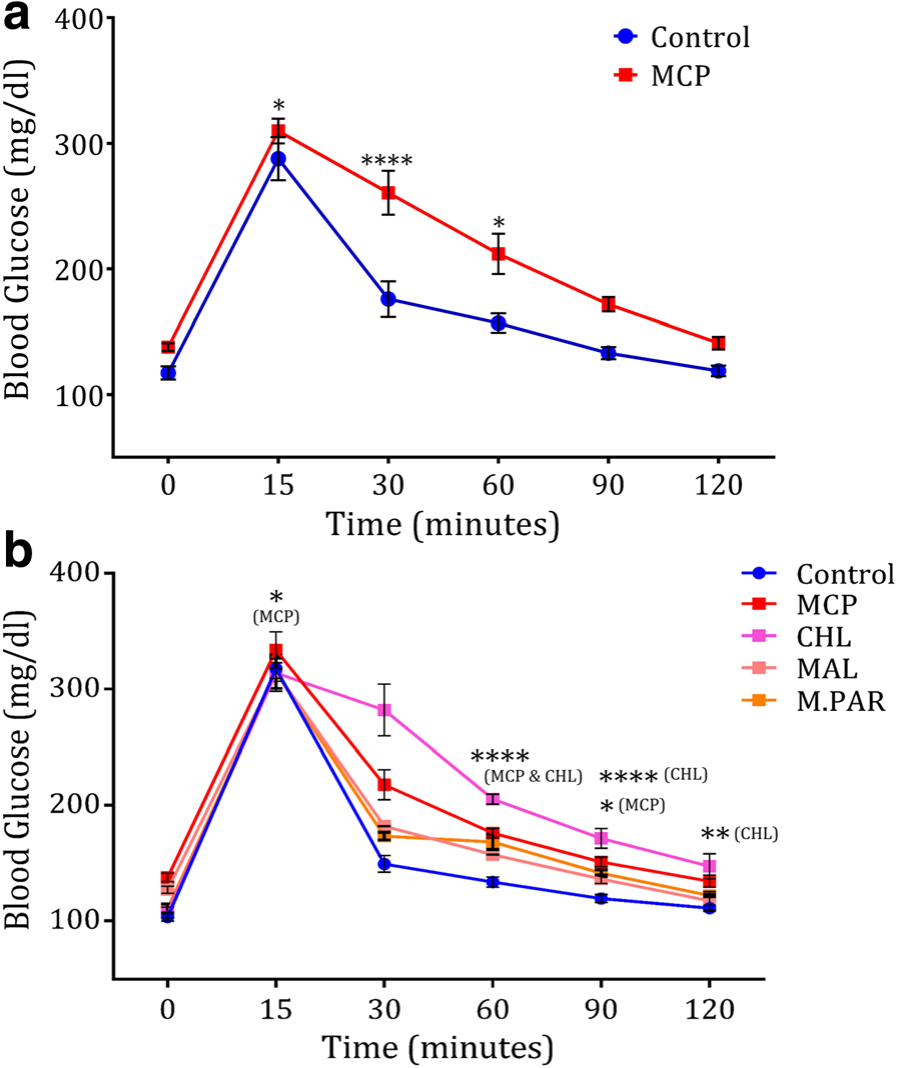
OP-induced glucose intolerance is mediated by gut microbiome **a** OGTT of animals following transplant of microbiota for seven days from pure water or MCP mixed water drinking mice (n = 08). **b** OGTT of animals following seven days of transplantation of fecal cultures grown in presence of OP (n = 06). *Horizontal lines* or *symbols* represent mean; *error bars* represent s.e.m; ****P < 0.0001, **P < 0.01, *P* < 0.05. Unpaired two-sided student-t test (**a**) or two-way ANOVA with Bonferroni correction (**b**). Experiments were repeated twice

AChE inhibition is the prime mode of action of OP [7] but in our study both humans and mice exposed to chronic OPs developed hyperglycemia but no changes in the level of plasma AChE activity is observed. OPs are biodegradable by the microbes [16] and xenobiotics were also reported to alter the gut microbiome and influence the physiology and pathology of mammals [22, 23]. Hence, in the present study we analyzed the influence of gut microbiome in OP-induced glucose dyshomeostasis. Fecal transplantation was executed in randomly selected animals for seven days using the fecal samples collected from control and MCP-fed animals (Additional file 1: Figure S9A). The animals were maintained in similar environments but in different chambers to avoid cross-contamination. The recipients of fecal microbiota from MCP-fed animals exhibited significant glucose intolerance on comparison to recipients of control microbiota (*P* < 0.05; Fig. 3a and Additional file 1: Figure S9B). The experiments were repeated twice and no significant variation between the batches were noticed (Additional file 4: Table S13).

To further check whether this induction of glucose intolerance is specific to MCP or common to all OP, feces from randomly selected mice were cultured anaerobically in the presence of MCP, CHL, MAL, or M.PAR and subsequently mice were fed with these cultures (Additional file 1: Figure S10A). This culture transplantation resulted in the replication of glucose intolerance condition (Fig. 3b). Except MAL (*P <* 0.60), all other pesticides MCP (*P <* 0.01), CHL (*P <* 0.0001), and M.PAR (*P <* 0.0003) caused significant glucose intolerance (Fig. 3b, Additional file 1: Figure S10B, and Additional file 4: Table S14), which is in concordance with the human data. Together, these results indicate that gut microbiome plays an inevitable role in the induction of glucose intolerance by OP and it seems to be a uniform mechanism among most of the OP.

### OP exposure induces OP-metabolizing machinery of gut microbiota

**Fig. 4:**
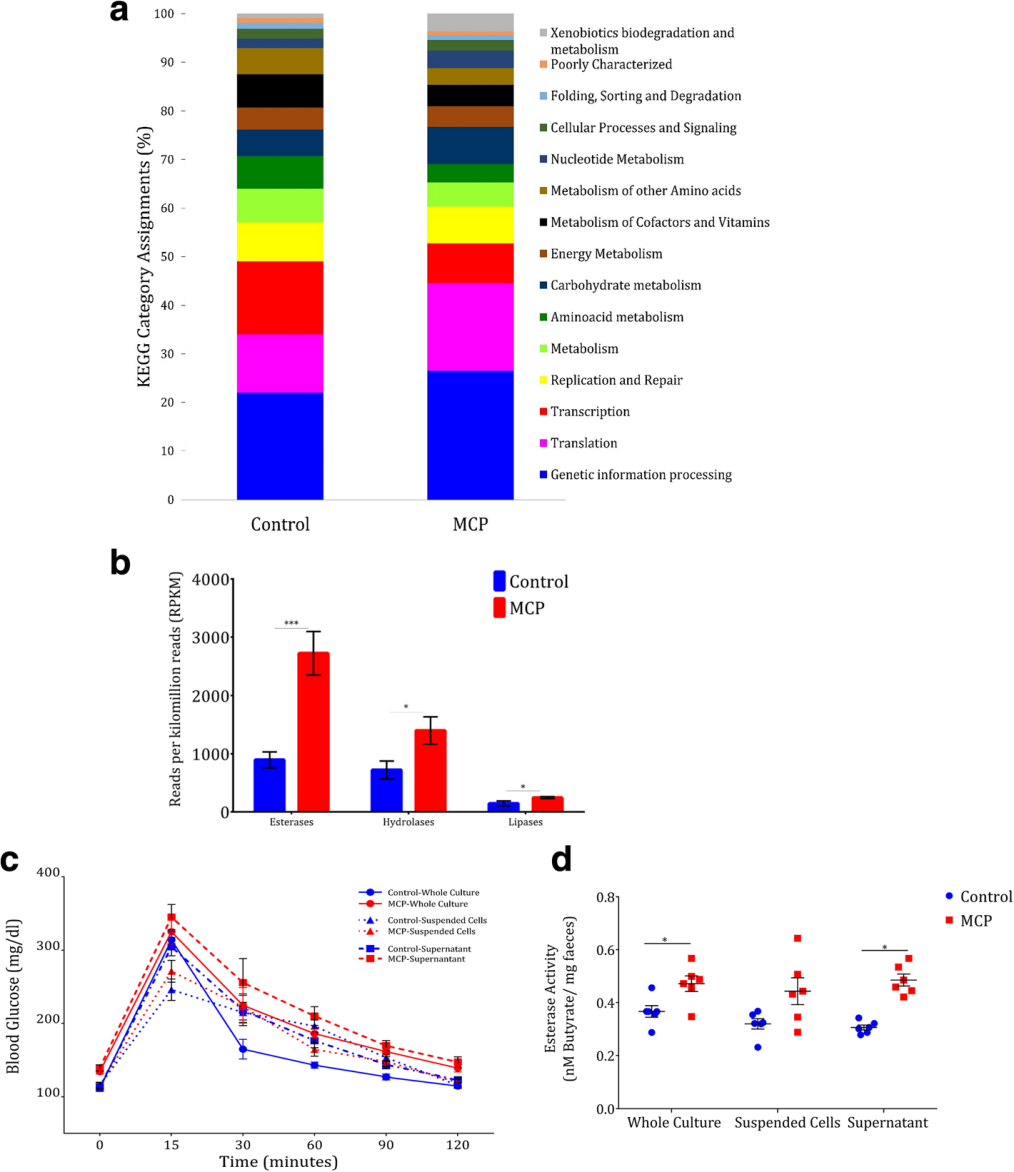
Chronic OP exposure activates the gut microbiome xenobiotic metabolism genes. **a** Percentage of normalized counts assigned to each KEGG category module. **b** Expression profile of OP degrading genes expressed as reads per kilomillion counts (RPKM) (n = 3). **c** OGTT of animals fed with fecal culture/suspended cells/supernatant grown in the presence or absence of MCP (n = 10). **d** Fecal esterase activity of the animals fed with fecal culture/suspended cells/supernatant grown in the presence or absence of MCP (n = 6). *Horizontal lines*, *bars*, or *symbols* represent mean; *error bars* represent s.e.m; ***P < 0.001, *P < 0.05. Unpaired two-sided student-*t* test (**b**, **d**) or two-way ANOVA with Bonferroni correction (**c**). Experiments were repeated twice

Antibiotics are known to shape the physiology and gene expression of the active human gut microbiome [22]. In order to understand the functional effects of OP on gut microbiome, we studied metatranscriptomics of bacterial RNA from the caecum of control and MCP-fed animals. Total RNA was isolated from caecum along with its content and the eukaryotic RNAs and bacterial ribosomal RNAs (rRNAs) were selectively removed and bacterial messenger RNA (mRNA) was enriched. RNA sequencing (RNA-seq) was performed yielding millions of reads which were annotated to the mice genome, human microbiome database (2012), and all other RNA libraries (Additional file 4: Table S15). To obtain a high-level view of the transcriptional response of OPs, the number of normalized counts assigned to each KEGG metabolic pathway were tallied. OP treatment resulted in increased expression of xenobiotic biodegradation and metabolism KEGG category. As previously reported in the case of antibiotic treatment [22], the expression of modules linked to genetic information processing, particularly translation, was increased while the transcription module was relatively decreased (Fig. 4a and Additional file 4: Table S16). Analysis of KEGG module and pathway abundance with HUMAnN and LEfSe confirmed and extended these trends: OPs induced the expression of modules for xenobiotic metabolism, glucose metabolism, phosphate transport, vitamin biosynthesis, nucleotide metabolism, and translation. Further on analysis with the metacyc enzyme database from the human microbiome consortium, we found a significantly increased expression of enzymes linked to OP degradation (Fig. 4b). These enzymes include esterases (*P* < 0.0005), hydrolases (*P* < 0.02), and lipases (*P* < 0.05) (Fig. 4b), which were characterized as potential OP degraders [16].

RNA-seq analysis indicated the upregulation of OP degrading bacterial enzymes during chronic exposure (Fig. 4b). To study its effect on glucose intolerance, the animals were fed with whole fecal cultures or phosphate buffered saline (PBS) suspended microbial cells or culture supernatant grown in presence and absence of MCP. Glucose intolerance was induced in animals receiving MCP culture (*P <* 0.03) and MCP supernatant (*P* < 0.04; Fig. 4c and Additional file 1: Figure S10C). In contrast, glucose intolerance was not induced in animals receiving MCP-suspended cells (Fig. 4c, Additional file 1: Figure S10C, and Additional file 4: Table S17). We also observed increased fecal esterase activity in the animals exhibiting impaired glucose tolerance phenotype (Fig. 4d, Additional file 1: Figure S10D, and Additional file 4: Tables S18 and S19). In sum, RNA-seq analysis and subsequent studies indicate that OP chronic exposure induces the expression of OP degradation machinery of the gut microbiome that appears to be the key mechanism behind impaired glucose tolerance.

### Acetic acid produced by microbial degradation of OP induces gluconeogenesis

**Fig. 5:**
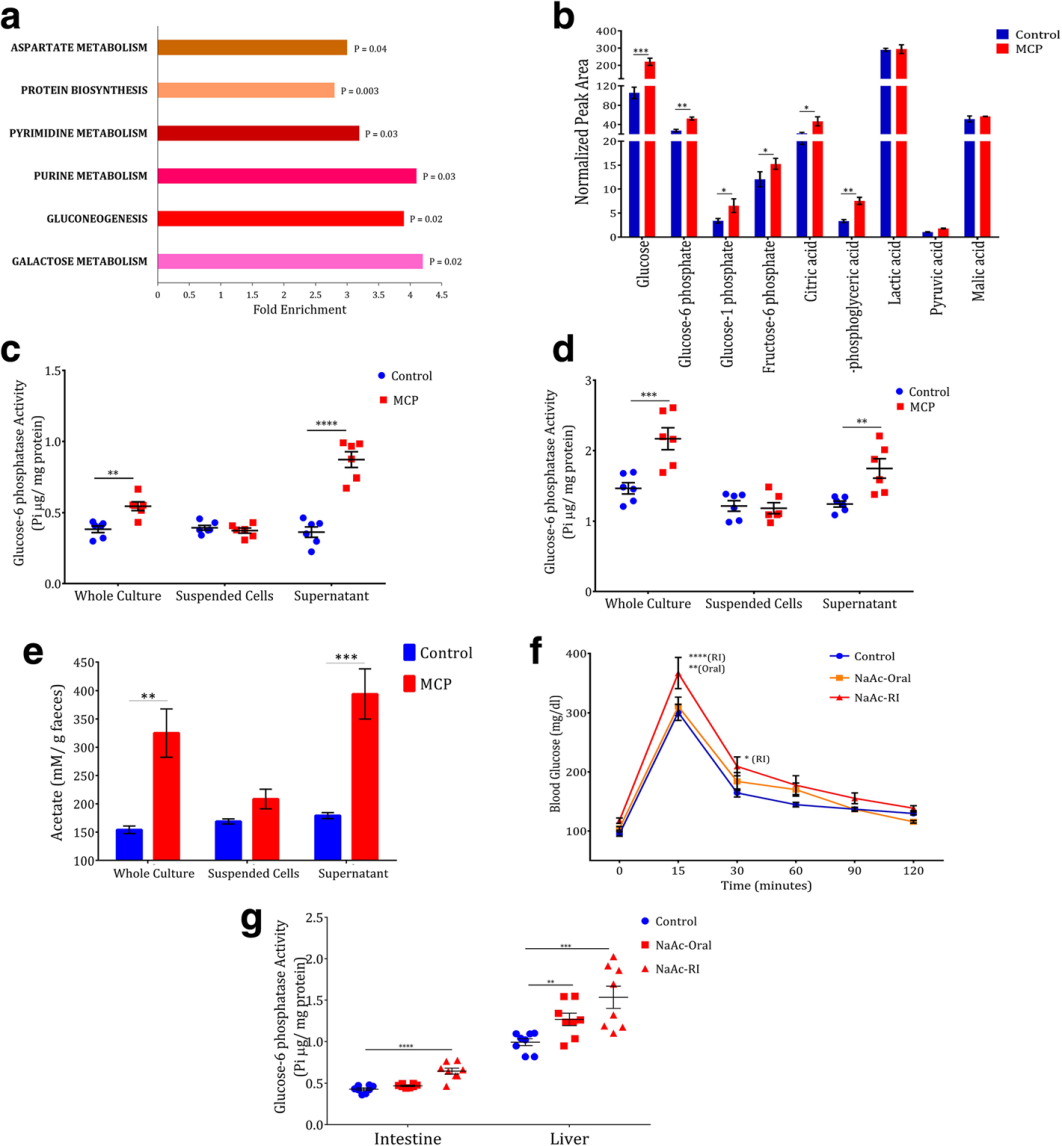
Gut microbial degradation of OP-induces gluconeogenesis. **a** Top five metabolic pathways represented by quantitative MSEA. The *P* values of Q-statistics are mentioned at the end of *bars*. **b** Expression of metabolites associated with gluconeogenesis expressed as normalized peak area (n = 3). **c** Intestinal and (**d**) Hepatic glucose-6 phosphatase activity of animals fed with fecal whole culture or suspended cells or culture supernatant grown in the presence or absence of MCP (n = 06). **e** Fecal acetate level of the animals fed with fecal whole culture or suspended cells or culture supernatant grown in the presence or absence of MCP (n = 03). **f** OGTT of animals treated with sodium acetate (NaAc) orally and by rectal infusion (RI) (n = 08). **g** Intestinal and hepatic glucose-6 phosphatase activity of NaAc treated animals (n = 08). *Bars*, *horizontal lines*, or *symbols* represent mean; *error bars* represent s.e.m; ****P < 0.0001, ***P < 0.001, **P < 0.01, *P < 0.05 Two-way ANOVA with Bonferroni correction (**f**) or one-way ANOVA with Tukey post-hoc analysis (**g**) or unpaired two-sided Student’s *t*-test (**b**–**e**). Experiments were repeated twice

To understand the effect of the microbial degradation of OP on host metabolism, we executed whole metabolite profiling of caecum tissue from control and MCP-fed animals. The expression of metabolites was expressed as peak area normalized to total ion chromatogram (Additional file 4: Table S20). Subsequently, the biologically meaningful pathways, which were significantly enriched in quantitative metabolomics data, were identified by quantitative metabolite set enrichment analysis (MSEA) by MetaboAnalyst [28] (Additional file 1: Figure S11 and Additional file 4: Table S21). Among the top enriched pathways, gluconeogenesis (GNG) showed a significantly higher fold change (*P* = 0.0208; Fig. 5a), which is associated with glucose intolerance. The expression profile of key metabolites associated with GNG showed increased expression, (Fig. 5b, Additional file 1: Figure S12, and Additional file 4: Table S20) suggesting the induction of GNG. In particular, a significantly elevated level of glucose (*P <* 0.002; Fig. 5b) suggested the enhancement of endogenous glucose production. In the present study, glucose-6 phosphate (*P <* 0.02; Fig. 5b) and citric acid (*P <* 0.04; Fig. 5b), which are crucial check points in the inter-regulation of glycolysis and GNG showed significantly elevation.

To confirm the induction of GNG, glucose-6 phosphatase (G6Pase) activity (a major regulatory enzyme in gluconeogenesis [29]) was assayed in the intestine and liver of the animals fed with control or MCP whole culture, suspended cells, and culture supernatant. Significantly higher intestinal G6Pase activity was observed in animals treated with MCP whole culture (*P <* 0.008) and MCP supernatant (*P <* 0.0001; Fig. 5c and Additional file 4: Table S22). Concurrently, significant GNG was also induced in the liver, which was evidenced by increased G6Pase activity in animals treated with MCP whole culture (*P <* 0.001) and in MCP-supernatant treated animals (*P <* 0.007; Fig. 5d and Additional file 4: Table S23). Expression of hepatic G6Pase is known to be involved in glycogenolysis, which is also associated with acute OP exposure [8] and glucose intolerance. Similar levels of liver glycogen were observed in all groups indicating that glycogenolysis was not induced by OP in our experiment (*P* < 0.08; Additional file 1: Figure S5E and Additional file 4: Table S24).

Short chain fatty acids (SCFA) especially acetic acid was produced during bacterial degradation of MCP [16]. Hence, we examined the level of fecal acetate in the animals fed with MCP or control whole culture, suspended cells, and supernatant. The feces from animals exhibiting impaired glucose tolerance showed significantly higher level of fecal acetate content (Fig. 5e). Similarly, the animals fed with cultures incubated in presence of other OPs also showed increased fecal acetate content (Additional file 1: Figure S10F). Thus, fecal acetate levels directly correlate with impaired glucose tolerance condition. This was highlighted by the increased G6Pase activity in liver (Fig. 5d).

To confirm whether the acetic acid produced by OP degradation is the key factor behind OP-induced gluconeogenesis, we treated the animals with sodium acetate (NaAc) orally or by rectal infusion (RI) (Additional file 1: Figure S13A) and examined for glucose intolerance. NaAc treatment replicated the impaired glucose tolerance phenotype induced by OPs (Fig. 5f and Additional file 4: Table S25). Though glucose intolerance was observed in oral treatment (*P <* 0.05), RI induced relatively significant glucose intolerance (*P <* 0.01; Fig. 5f and S12B). We noticed significant intestinal G6Pase activity in animals rectally infused with NaAc (*P <* 0.0001; Fig. 5 g), but not in the animals orally treated with NaAc (*P <* 0.07; Additional file 4: Table S26). In contrast, significant hepatic G6Pase activity was observed in both groups of animals treated with NaAc orally (*P <* 0.008) or by RI (*P <* 0.001; Fig. 5 g and Additional file 4: Table S27). Exceptionally, increased glycogen content was observed in animals rectally infused with NaAc (*P <* 0.05; Additional file 1: Figure S13D and Additional file 4: Table S28). Collectively, these results indicate that acetic acid was produced by the gut microbial degradation of OP and thereby gluconeogenesis was induced in intestine and liver.

### Fecal acetate and esterase activity correlates with OP residues and human diabetic status

To examine the prevalence of a similar phenomenon of gut microbiota-dependent OP-induced hyperglycemia in humans, we collected fecal samples from control (n = 60) and diabetic people (n = 60) in the same population that studied for OP residues (Additional file 2: Table S8). Pregnant women and those having stomach/bowel-related disorders were excluded from the study. Among the non-diabetics, people with obesity, hypertension, hypocholesterolemia or any other self-reported disorder, and those on any kind of regular medications were excluded from the study and thereby only control people were included.

**Fig. 6:**
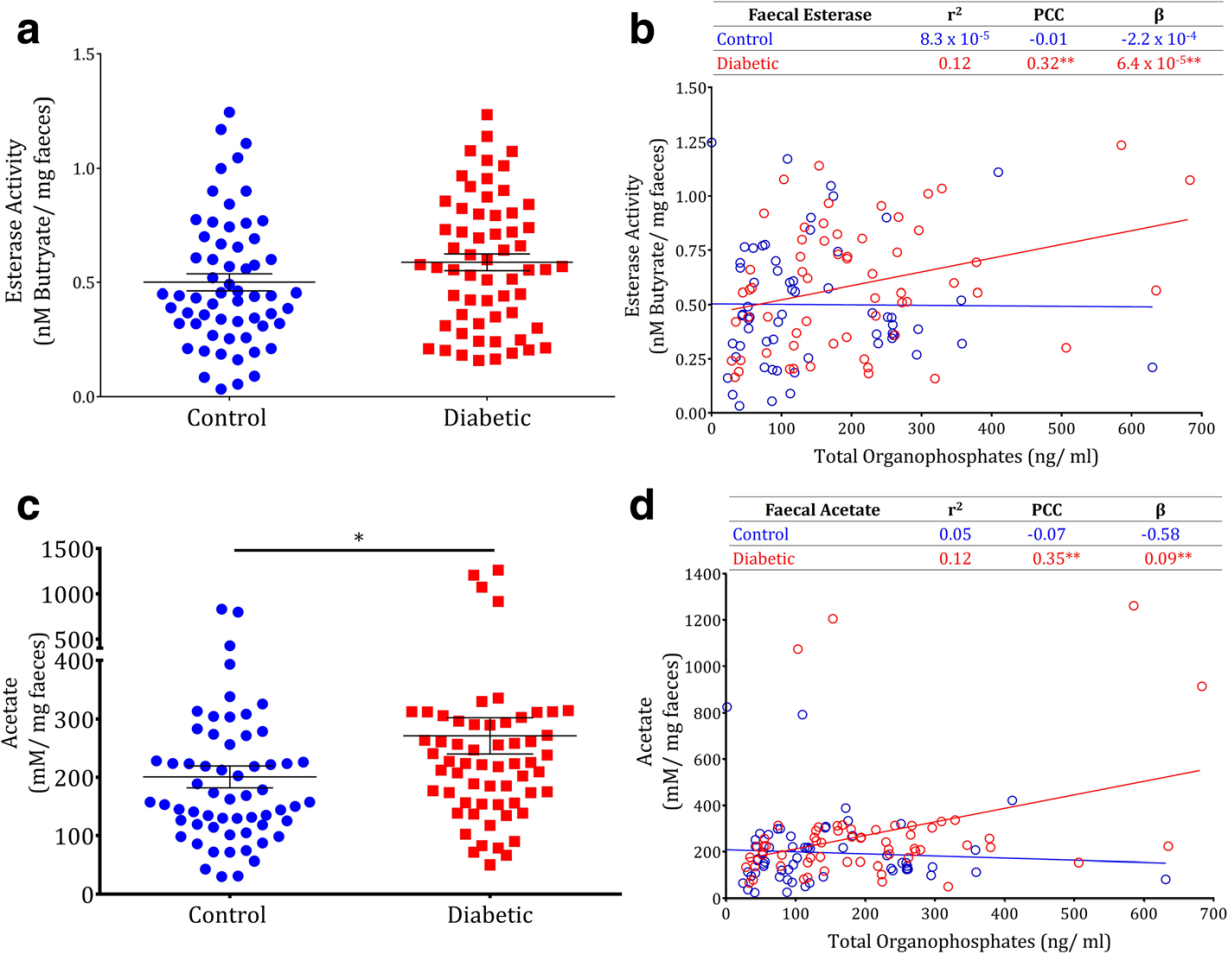
Plasma OP residues correlate with fecal esterase activity and fecal acetate. Fecal samples were collected from control (n = 60) and diabetic (n = 60) humans. **a** Fecal esterase activity of non-diabetic vs. diabetic individuals. **b** *Regression plot* of plasma total OPs vs. fecal esterase activity. **c** Fecal acetate level of non-diabetic vs. diabetic. **d** *Regression plot* of plasma total OPs vs. fecal acetate content. *Horizontal lines* represent mean; *error bars* represent s.e.m; *P < 0.05 Rank sum, Mann–Whitney U Test (**a**, **c**). The *hollow* circle represents individual values and *straight line* represents the trend line. *PCC* Pearson correlation coefficient, *β* regression coefficient. *P < 0.05; **P < 0.01

We applied esterase assay to study the OP degrading potential of fecal microbiota from diabetic and control individuals. No significant association between fecal esterase activity and diabetic status (Rank sum *P <* 0.40; Fig. 6a) was observed. However, positive correlation was obtained between OP residues and fecal esterase activity (PCC = 0.32, *P* > 0.01) (Additional file 2: Table S9). On regression analysis, we observed a linear trend in the increase in fecal esterase activity for every unit increase of total OP in plasma of diabetic individuals (β = 6.4 × 10^−4^, *P* > 0.01) (Fig. 6b). Subsequently, we checked for the fecal acetate level by gas chromatography and found a significantly higher acetate level among the diabetic people (Rank sum *P >* 0.03; Fig. 6c and Additional file 1: Figure S14). As observed for esterase activity, significant correlation and linear trend were observed between total OP and acetate level in the diabetic people (PCC = 0.35, *P* > 0.01; β = 0.09, *P* > 0.01) (Fig. 6d). In the case of MAL, a significant positive correlation (PCC = 0.18, *P* > 0.05) was obtained for esterase activity but no correlation was found for acetate (PCC = 0.03, *P* = 0.06) (Additional file 2: Table S9). On interquartile analysis, the people in the highest quartile are largely distributed among the diabetic population (Additional file 1: Figure S14). Overall, these results suggest the prevalence of a similar phenomenon of diabetic conditions mediated by microbial degradation of OPs in humans.

## Discussion

In the last few decades, the epidemic of diabetes intensified concurrently with increased consumption of synthetic chemicals including OPs [30]. We detected a probable link between direct exposure to OPs and self-reported diabetic status in a rural population (Fig. 1a). Previous reports also showcased OPs with increased odds of diabetes in a dose-dependent manner [12, 14]. As periodical health camps are conducted by our University and Government in the study villages, most of them are aware of their diabetic status. In concurrence with previous reports [14, 15], we noticed that diabetes was highly prevalent among the rural people directly exposed to OPs (Fig. 1a and Additional file 1: Figure S1B), who are majorly agricultural workers involving huge physical work. A prevalence of nearly 50% of non-genetic diabetics in both groups suggests that genetic risk may not be the causative factor for increased prevalence of diabetes among the directly OP exposed group (Additional file 1: Figure S1C).

To further validate the self-reported diabetes and OP exposure, blood samples were collected. Though 10% of self-reported diabetic people had normal glucose levels, they were classed as diabetic since they were taking hypoglycemic medication (Additional file 2: Table S4). On validation of self-reported diabetes, we found 19% of self-reported non-diabetics were newly diagnosed as diabetic (Additional file 2: Table S4), which adds to the burden of diabetic prevalence in this community. The major mode of action of OP is inhibition of AChE but we found no significant changes in plasma AChE activity with either OP exposure or HbA_1c_. The people with elevated AChE levels were evenly distributed between the diabetic and non-diabetic individuals and no correlation was found with plasma OP residues (Fig. 1b, Additional file 1: Figure S2A, and Additional file 2: Table S4). Any changes in AChE activity, neuronal markers, or prevalence of neuronal diseases were not described in the previous epidemiological studies which report the association between OP exposure and diabetic incidence [12–15]. The OP residues detected in the people not directly exposed (Fig. 1c–e, Additional file 2: Table S4) indicates the draining of the OPs from farm to the public *via* air, water, and eatables. Though people are majorly exposed to OPs *via* food, the OPs in the small intestine enter the blood stream and re-enter the large intestine through bile excretion. The linear trend between OP residues and HbA_1c_ shows the direct or indirect role of OPs in the diabetic epidemic in the past few centuries. Except MAL, all the other OPs showed a significant correlation and regression with HbA_1c_ values. MAL was categorized under WHO Class III (slightly hazardous) while the rest of them are under Classes I, IIa, and IIb (extremely, highly, and moderately hazardous, respectively) (Additional file 2: Table S1).

To check the diabetogenic nature of OPs, animals treated with 10× TMDI dose of OPs for a chronic time period resulted in significant glucose intolerance (Fig. 2a and c) and oxidative stress (Fig. 2d and Additional file 1: Figure S8) leading to hepatic damage (Additional file 1: Figures S7F and S8E) with no change in AChE activity (Fig. 2b). TMDI calculation does not include the vegetables with no MRL value, drinking water, air, soft drinks, snacks, and other consumables. Hence, we provided the animals with 10× TMDI dose, which is 47.2 times (Additional file 1: Figure S7A) higher than the acceptable daily intake (FAO/WHO, 1996). In the absence of AChE inhibition during chronic exposure, the mechanism of OP-induced glucose dyshomeostasis is not clearly defined [8, 9].

The biodegradable nature of OPs made them an acceptable alternative to persistent organochlorines [3]. Recent studies showcased the role of gut microbiome in the action of drugs and other chemicals [31]. Fecal transplantation from MCP-fed animals induced glucose intolerance (Fig. 3a and Additional file 1: Figure S8B) and this phenotype was reproduced in animals fed with fecal cultures grown in the presence of other OPs except MAL (Fig. 3b and Additional file 1: Figure S9B). This is in concordance with previous reports that MAL induces a gradual increase in blood glucose followed by decrease in blood glucose that can even reach hypoglycemia [32]. Chronic OP exposure activates the expression of OP metabolizing genes of the gut microbiome (Fig. 2b) and subsequent OP degradation produces acetic acid. These OP degrading enzymes are well characterized enzymes and employed as potential players in OP remediation projects [16]. Though the fecal cultures grown in the presence of OPs and their supernatants could induce glucose intolerance, the microbial cell suspension was not able to induce the same (Fig. 4c). A similar trend was observed in esterase activity (Fig. 4d) and this indicates that byproducts of OP degradation are able to induce glucose intolerance but the modified microbiome with degrading potential lacks this property. Though the microbes in cellular suspension have OP metabolizing potential, they do not have the substrate OPs to produce acetate and hence glucose intolerance was not induced. While in the case of whole culture and supernatant, the by-products of OP metabolization, including acetate, were present, which acts as a substrate for gluconeogenesis and thereby glucose intolerance was induced.

Metabolomic analysis clearly showcased the induction of GNG (Fig. 5a and b). Intestinal GNG was shown to induce benefits in glucose control since it initiates a neutrally mediated suppression of hepatic glucose production [33]. However, when hepatic GNG is strongly altered, e.g. when bile salts are released in the portal blood, hepatic GNG dominates the regulatory action of intestinal GNG [34]. Hence, due to the maximal activation of hepatic GNG, the protective effect of intestinal GNG is not predominant in the effects of OP. Though the liver is the primary site of GNG, this pathway is also induced in the intestine and kidneys during specific conditions. Intestinal GNG is a central signal in glucose and energy homeostasis [33]. We observed no significant change in the expression levels of glucogenic amino acids, which indicates GNG is not induced by a substrate effect putatively initiated by these amino acids as substrates (Additional file 1: Figure S8). Glycogenolysis is another potential pathway by which glucose is produced from liver glycogen using GPase. OPs such as MPAR, MAL, and acephate were reported to induce glycogenolysis and decrease liver glycogen content in animal experiments at acute and subchronic exposure [8]. However, in our study no significant change in liver glycogen level was observed. This indicates that the mechanism behind OP-induced glucose dyshomeostasis may vary between acute and chronic toxicity. A pentose phosphate pathway is yet another metabolic network that regulates glucose homeostasis but no significant changes were observed in the level of metabolites linked to this pathway in our analysis (Additional file 2: Table S16) and this is inconsistent with the previous reports. SCFA are produced during microbial degradation of OPs [16]. Among the SCFA, only butyrate rather than other SCFAs is completely utilized in the intestine [35], which is not produced by the degradation of any OPs [16]. Microbiota are proven to regulate intestinal absorption and metabolism of fatty acids. While regarding other SCFAs, especially acetate, only a small fraction is utilized in the intestine and the remaining reaches the liver *via* the periportal vein [36] and hepatic GNG is activated. A linear regression between OP residues and fecal esterase activity indicates the induction of the expression of OP degrading genes in OP environment and this is evidenced by the increased fecal acetate observed in the diabetic samples. Thus, the association between human diabetes and fecal esterase activity and fecal acetate with plasma OP residues signs the probable prevalence of gut-microbiota mediated OP-induced hyperglycemia in humans (Fig. 6).

## Conclusion

**Fig. 7:**
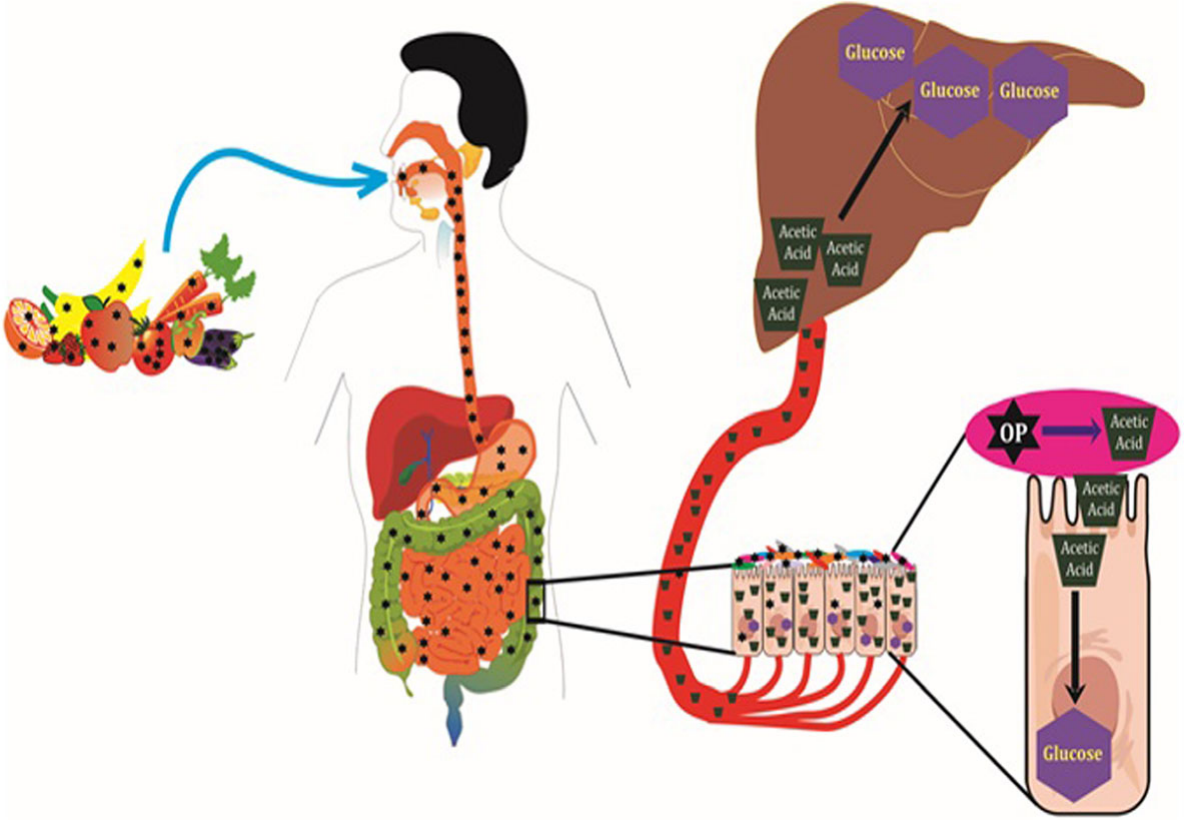
*Schematic summary* of the molecular mechanism behind gut microbiome mediated OP-induced glucose intolerance. OPs (*star*) enter the human digestive system *via* food and are metabolized into acetic acid (*trapezoid*) by the gut microbiota (*oval*). Subsequently, acetic acid was absorbed by the intestinal cells and the majority of them were transported to the liver through the periportal vein. Eventually, acetic acid was converted into glucose (*hexagon*) by gluconeogenesis in the intestine and liver and thus accounts for glucose intolerance

In summary, our study showcased a probable association between plasma OP residues and diabetes with no significant changes in plasma AChE. OPs are metabolized by the gut microbiome to acetic acid, which is utilized as the substrate for GNG and accounts for glucose intolerance (Fig. 7). Today the words of Carson [2], “As the tide of chemicals born of the industrial age arisen to engulf our environment, a drastic change has come about in the nature of the most serious health problem,” became invincible truth. Our study revealed that gut microbiome-mediated metabolism of OPs could be a key risk factor for diabetes and thereby calls for the reconsideration of OP usage all over the world. The observance of correlation of OP residues with HbA_1c_ and fecal parameters suggests the establishment of environmental chemicals and gut microbiota as diagnostic markers and therapeutic targets for metabolic diseases. In fact, OPs were once considered as a better alternative to the persistent organochlorines but today it appears that detailed experiments on the toxicity of these non-persistent pesticides is needed. Hence, rather than searching for other chemical alternatives, promotion and development of traditional self-sustainable, nature-based agricultural practices would be the right approach to feed this world.

## Methods

### Human survey

A survey was conducted among the pesticide shop workers, pesticide applicators, and land owners in Vadapalanji Panchayat Union, Madurai district, Tamil Nadu, India (latitude 9.9272, longitude 78.0092; Additional file 1: Figure S1). Details on name, frequency, and volume of insecticides commonly being used were collected. The fungicides, herbicides, bactericides, and mineral supplements were not taken into consideration. In a subsequent study, the people (n = 3080) from villages in Vadapalanji Panchayat Union that incorporates Vadapalanji, Manapatti, Thenpalanji, Sundarajapuram, Nagamalai puthur and Palkalainagar, Vellaparaipatti, and Meenatchipatti villages of Madurai district, Tamil Nadu, India were surveyed for their diabetic status and exposure history to OPs using a questionnaire (Additional file 3). Participants under the age of 35 years and pregnant women were excluded from the study. In addition, sex, age, and familial diabetic history were also collected. The diabetic status of the participants was self-reported by answering “Yes” or “No.” Participants involved in OP spraying, mixing, and field workers in OP-based agriculture were defined as having direct exposure to OPs and those who were not associated with OP-based agricultural works were defined as having indirect exposure to OPs.

### Human blood collection

Blood samples of 5 mL were collected in EDTA-coated tubes from a random subset of the population (n = 802) involved in an earlier survey. The details of age, sex, height, weight, occupation, dietary habits, smoking, alcohol and tobacco consumption, exposure history to pesticides, prevalence of diabetes or any other diseases, and familial diabetic history were collected using a standard questionnaire (Additional file 5). Obesity was defined as body mass index (BMI) > 30 kg/m^2^. The collection protocol was approved by the internal research and review board, ethical clearance, biosafety, and animal welfare committee of Madurai Kamaraj University. Laboratory personnel performing analyses of blood parameters were all blinded and provided with only the sample ID and no participant details.

### HbA_1c_ analysis

Diabetic status was confirmed by HbA_1c_ analysis in whole blood by HPLC (D10, Biorad Inc., USA). Diabetes was defined as either having a history of diabetes on medications or glycated hemoglobin (HbA_1c_) of ≥ 6.5% based on International Expert Committee (IEC) recommendations.

### Acetylcholine esterase assay

AChE assay was performed in blood plasma/serum using the Amplex Red acetylcholinesterase kit (Invitrogen Inc., USA; A12217) as per the manufacturer’s instructions. In this assay, AChE activity is monitored indirectly using 10-acetyl-3,7-dihydroxyphenoxazine (Amplex Red), a sensitive fluorogenic probe for hydrogen peroxide. First, AChE converts the acetylcholine substrate to choline, which is oxidized by choline oxidase to betaine and H_2_O_2_. The latter, in the presence of horseradish peroxidase, reacts with Amplex Red in a 1:1 stoichiometry and generates a highly fluorescent product, resorufin [37]. The fluorescence was measured using excitation in the range of 545 nm and emission at 590 nm. The reactions were performed with technical triplicates. The reference values are 3334–7031 mU/L for males and 2504–6297 mU/L for females.

### OP residues analysis by GC/MS

Blood plasma samples were extracted by a dispersive liquid–liquid microextraction technique [38] by a modified method. Briefly, 200 μL of plasma sample was spiked with 1 mg/mL of azobenzene as internal standard followed by the addition of 20 μL of 5 N HCl and made up to 1 mL with deionized water. Subsequently, the sample is incubated at 70 °C for 30 min to avoid the interaction of OPs with proteins. After cooling down to room temperature, 150 μL of acetonitrile (dispersive solvent) and 50 μL of chloroform (extraction solvent) mixture was forcibly added to the sample using syringe and sonicated for 3 min followed by centrifugation at 10,000 rpm for 5 min. The organic phase at the bottom of the tube is carefully collected and dried under a gentle stream of nitrogen gas and dissolved in 20 μL of hexane. Sample preparation and analysis were done in a blinded fashion.

Primary stock solutions of each insecticide (1 mg/mL) were prepared in methanol. Working standard solutions of the compounds were prepared by combining the aliquots of each primary solution and diluting with hexane. The stock solutions were stored at −20 °C in the dark when not in use. The standards were run at different concentrations and peak area was observed and subsequently linearity was established. Limit of detection (LOD) and limit of quantification (LOQ) were determined by standard methods. The recovery efficiencies for each individual insecticide was determined by spiking known concentrations of insecticide and measurement by GC/MS (Clarus 680/ 600C; Perkin Elmer Inc., USA).

The GC injector temperature was set at 200 °C. The oven temperature program was optimized to hold at 120 °C for 1 min and then to increase by 10 °C min^−1^ up to 220 °C [39]. Helium gas was used as carrier gas. The transfer line temperature was adjusted to 280 °C. Mass spectrometry conditions were as follows: electron ionization source set to 70 eV, emission current 500 lA, MS Quad 150 C, MS Source 200 °C. The mass spectrometer was run in full-scan mode and in single ion monitoring mode. The m/z fragments monitored for each insecticide is provided in Additional file 1: Figure S2C.

### Maintenance and breeding of animals

Mice of *BALB/c* strain (RRID:IMSR_HAR:1255) were obtained from Madras University, Chennai and maintained and bred in an animal house at 25–28 °C with 12 h day/night cycles. The animals were fed with deionized water and standard rat chow (Hindustan Lever Limited, India) ab libitum. All the experiments in this study were performed with female mice aged eight weeks weighing 20–28 g and maintained in a constant environment at 25–28 °C with 45–60% humidity. The health status of the mice was confirmed by continuously monitoring their activities, behavior, body weight, and feces. The animal protocols used in this study were approved by the internal research and review board, ethical clearance, biosafety, and animal welfare committee of Madurai Kamaraj University.

### Administration of monocrotophos to animals

The mice were provided MCP (Sigma-Aldrich Inc., USA; 361173) at 10× TMDI dose [6] (28 μg/kg bodyweight/day) directly in drinking water for 180 days. The food and liquid intake and animal activity were monitored daily. The body weight of the animals was documented every 30 days.

### Measurement of fasting blood glucose

The animals were allowed for overnight fasting prior to blood glucose measurement. The fasting glucose was measured using a biosensor based glucometer [40] (Johnson & Johnson Inc., USA; OneTouch) with a drop of blood from the tail vein and expressed as mg/dL.

### Oral glucose tolerance test

OGTT was performed to analyze how quickly the glucose is cleared from the blood [41]. The animals were fasted overnight prior to execution of OGTT, blood was sampled by the tail vein, and glucose was measured using the glucometer (0 min). Subsequently, the animals were gavaged with glucose solution (1.5 g/kg body weight) and blood glucose was monitored at 15, 30, 60, 90, and 120 min. The data were plotted in blood glucose versus time curve and glycemic response was expressed as area under the curve (AUC, × 10^3^).

### Harvest of organs

The animals were anesthetized by subcutaneous injection of ketamine (100 mg/kg body weight). The blood was collected by cardiac puncture and the organs including the brain, heart, liver, kidneys, and large intestine were harvested and perfused in sterile PBS (10× g/ l: 25.6 Na_2_HPO_4,_ 80 NaCl, 2.0 KCl, 2.0 KH_2_PO_4_. pH 7.2) and stored at −80 °C.

### Serum insulin

Insulin level in the serum was determined by immunoenzymometric assay based kit (Monobind Inc., USA; 5825–300) as per the manufacturer’s instructions.

### Preparation of tissue homogenate

A total of 100 mg of the tissue was homogenized in 1 mL RIPA buffer (Sigma-Aldrich Inc., USA; R0278), supplemented with 100 μL of cocktail protease inhibitors (Sigma-Aldrich Inc., USA; P8340), and incubated in ice for 20 min. The homogenate was centrifuged at 12,000 rpm for 20 min at 4 °C and the supernatant was collected, aliquoted, and stored at −80 °C.

### Protein estimation

The amount of protein in serum/tissue homogenate was estimated by Bradford assay [42] (Sigma-Aldrich Inc., USA; B6926) as per the manufacturer’s instructions. Bovine serum albumin was used as a standard and the reactions were performed with technical triplicates.

### Protein carbonylation assay

The carbonyls produced by oxidation of protein measured spectrophotometrically by the dinitrophenyl hydrazine (DNPH) method [43]. Briefly, 100 μL of the serum/tissue homogenate were mixed with 400 μL of 10 mM DNPH dissolved in 2.5 M HCl and incubated for 60 min and the protein was precipitated with an equal volume of trichloroacetic acid (TCA) (10%). The resultant pellet was washed with 1:1 ethanol:ethyl acetate mixture and resuspended in 250 μL of 6 M guanidine HCl. The protein hydrozones were measured spectrophotometrically at 370 nm. The corrected absorbance (CA) for each sample was calculated by the difference between corresponding control. The concentration of protein carbonyls (nM) was determined as follows: ((CA)/0.011) (250/100)). The reactions were performed with technical triplicates.

### Lipid peroxidation assay

The lipid peroxidation was determined by estimating malondialdehyde (MDA) [44] with minor modifications. Briefly, 100 μL of tissue homogenate was added with 200 μL of ice cold 10% TCA to precipitate the protein and kept in ice for 15 min. After incubation, the samples were centrifuged at 2200 rpm for 15 min at 4 °C. A total of 200 μL of supernatant was added with equal volume of 0.67% thiobarbituric acid (TBA) and then incubated in a boiling water bath for 10 min. 1,1,3,3’-tetramethoxypropane was used as the standard. The color developed was read at 532 nm and the amount of MDA was expressed as nM/mg protein. The reactions were performed with technical triplicates.

### Total antioxidant assay

The total antioxidant assay in the serum was executed using the total antioxidant kit (Sigma-Aldrich Inc., USA; CS0790) as per the manufacturer’s instructions. The principle of this assay is the formation of a ferryl myogloblin radical from metmyoglobin and hydrogen peroxide, which oxidizes the ABTS (2,2’-azino-bis(3-ethylbenzthiazoline-6-sulfonic acid) to produce a radical cation, ABTS^•+^, a soluble green chromogen that can be determined spectrophotometrically at 405 nm [45]. Trolox, a water-soluble vitamin-E analog, serves as the standard. The reactions were performed with technical triplicates. The antioxidant concentration was expressed in mM relative to the concentration of the Trolox standard.

### Histopathology

The perfused hepatic tissue was fixed with 10% formaldehyde and paraffin embedded by standard methods. The embedded tissues were sliced into 5-μm thin sections using rotatory microtome. The sections were stained with hematoxylin and eosin and mounted on slides. Finally, the tissue morphology was examined by light microscopy, recorded, and analyzed by a qualified clinical pathologist in blinded fashion.

### Fecal transplantation

After 180 days of experiment, 200 mg of the fecal material from animals drinking pure water or MCP mixed water was collected and suspended in 5 mL of PBS, mixed and incubated for 5 min at room temperature for separation by gravity, and the upper phase was collected. The mice were randomly selected for the study and allowed for 4 h fasting before fecal transplantation. Mice were gavaged with 200 μL of suspension per day for seven consecutive days [27]. The mice gavaged with fecal suspension were maintained in a separate, adjacent glass chambers to avoid cross-contamination. Similar conditions of temperature, humidity, water, and feed were maintained between the control and fecal transplanted group. At the end of the week, an OGTT was conducted as described above. Twenty-four hours after OGTT, the mice were sacrificed and the intestine and liver were collected for other assays.

### Ex vivo culture and feeding

A total of 200 mg of fecal content from randomly selected mice from different cages were collected and suspended in 5 mL of sterile PBS and vortexed. The mixture was allowed to stand at room temperature for 5 min for separation by gravity and the supernatant was collected. One milliliter of the supernatant was inoculated in 9 mL of Robertson cooked meat medium (composition g/L: beef heart solids 98; proteose peptone 20; dextrose 2; sodium chloride 5. pH 7.2) supplemented with different OPs (MCP, CHL, MAL, and M.PAR) (Sigma-Aldrich Inc., USA; 36173, 45395, 36143, 36187) at 0.2 mg/mL concentration and incubated at anaerobic condition at 37 °C for nine days [22, 27]. To maintain logarithmic growth, the culture was subcultured every three days. After nine days of growth, part of the culture was centrifuged at 3000 rpm for 5 min and the supernatant was collected while the remaining pellet was dissolved in same volume of PBS. As mentioned above in the fecal transplantation protocol, the mice were administered with 200 μL of whole culture/suspended cells/supernatant continuously for seven days and finally OGTT was performed. As described above, the mice fed with cultures were maintained in separate, adjacent glass chambers with the same environment of temperature, humidity, water, and feed. Twenty-four hours after OGTT, the mice were sacrificed and the intestine and liver were collected for other assays.

### Metagenomic RNA isolation

Total RNA was extracted from the ceacal tissue along with its contents using TRI reagent (Sigma-Aldrich Inc., USA; T9424) as per the manufacturer’s instructions. The integrity was checked in the agarose gel and quality and quantity was determined spectrophotometrically.

### Enrichment of bacterial RNA

Bacterial RNA was enriched from the total RNA by using MICROBEnrich kit (Ambion Inc., USA; AM1901) as per the manufacturer’s protocol. Here, hybridization capture technology was used to remove human, mouse, and rat RNA (both mRNA and rRNA) from complex host-bacterial RNA populations, leaving behind enriched microbial total RNA. In the first step of the procedure, host-bacterial total RNA is incubated with a mixture of capture oligonucleotides that bind the mammalian 18S and 28S rRNAs and polyadenylated RNAs. Next, the rRNA/oligo nucleotide hybrids and all polyadenylated mRNAs are removed from the mixture with oligonucleotide-derivatized magnetic beads. To ensure complete removal of eukaryotic mRNAs, complementary DNA was constructed with oligo-d(T) primers and polymerase chain reaction for the mouse GAPDH gene was executed and checked.

### Enrichment of bacterial mRNA

Bacterial mRNA was enriched in the purified RNA by removing the 16S and 23S rRNAs using a MICROBExpress kit (Ambion Inc., USA; AM1905) as per the manufacturer’s instructions. The method employs a modification to sandwich capture hybridization protocols that were developed for the capture and detection of specific nucleic acid molecules with probes conjugated to magnetic beads. The bound RNA was separated by using magnetic field and the unbound RNA was dissolved in RNase free water. The enrichment of bacterial mRNAs and removal of rRNAs was confirmed by bioanalyzer (Agilent Inc., USA) analysis.

### RNA sequencing and analysis

RNA library was constructed using TruSeq kit (Illumina Inc., USA) as per the manufacturer’s instructions. RNA-seq was done at Centre for Cellular & Molecular Platforms (Government of India), Bangalore with paired-end reads in Illumina HiSeq 1000 machine. The sequencing was performed in a blinded way. Raw data were processed using the Solexa software. Low-quality reads were filtered according to the base quality value. The reads were mapped with mouse genome, murine mRNAs, transfer RNAs, and rRNAs by Bowtie 2 [46] and the annotated sequences were removed.

We used a reference database of human microbiome to perform functional analysis of the RNA-seq data. This reference included 538 draft and finished bacterial genomes from the human microbiome consortium. High-quality reads were mapped using Bowtie 2 to our reference bacterial database. Subsequently, using the KEGG database, all predicted proteins from the reference genome database were annotated with KEGG orthologous groups (KOs). For query genes with multiple matches, the annotated reference gene with the lowest e value was used. When multiple annotated genes with an identical e value were encountered after a BLAST query, we included all KOs assigned to those genes. The number of transcripts assigned to each gene was then tallied and normalized to RPKM. To account for genes that were not detected owing to limited sequencing depth, a pseudocount of 0.01 was added to all samples. Genes were grouped by taxa, genomes, and KO by calculating the cumulative RPKM for each sample. HUMAnN [47] was used for metabolic reconstruction from metagenomic data followed by LefSe [48] analysis with bootstraping to identify significant biomarkers. The reads were annotated to metacyc enzyme database from the human microbiome consortium using BLASTN. The number of transcripts assigned to each enzyme were then tallied and normalized to RPKM. The enzymes of the same class were summed and expressed as single enzyme.

### Esterase assay

A total of 200 μL of the culture was centrifuged at 12,000 × rpm for 10 min and the pellet was suspended in 200 μL of sterile PBS. Eighty microliters of suspension were used for esterase assay with ethyl butyrate as substrate as per Lisboa et al. [49]. The formation of carboxylic acid due to hydrolysis of substrates mediated by esterase causes a reduction in the pH, which changes the color of the medium from blue to yellow. This reaction can be observed or monitored spectrophotometrically at 616 nm. We used ethyl butyrate (Sigma Aldrich Inc., USA; 109959) as the substrate and bromothymol blue (Himedia labs, India; GRM120) as the pH indictor.

### Metabolomics

The tissue processing for metabolomics was carried out in NIH Centre for Metabolomics, University of California, USA as per standard operating procedure [50]. The analysts were blinded of the sample information. A total of 50 mg of caecum tissue cleared of fecal matter was taken in a 25-mL polypropylene centrifuge tube and 2.5 mL of extraction solvent (acetonitrile:isopropanol:water 3:3:2) was added and homogenized for 45 s. In between every homogenization, the homogenizer was cleaned with solutions of methanol, acetone, water, and the extraction solvent. The homogenate was centrifuged at 2500 rpm for 5 min. The supernatant was aliquoted 2 × 500 μL and one of them stored at −20 °C for back up. The other aliquot of 500 μL was evaporated to complete dryness in a centrivap cold trap concentrator. The dried aliquot was resuspended in 500 μL of degassed 50% acetonitrile and centrifuged for 2 min at 14,000 rcf. The supernatant was collected in a fresh tube and evaporated to dryness in a centrivap cold trap concentrator and finally submitted to derivatization.

### Primary metabolism by ALEX-CIS GCTOF MS

Data were acquired using the following chromatographic parameters as described by Fiehn et al. [51]. A Rtx-5Sil MS column (Restek Corporation) was used with helium as a mobile phase. A total of 0.5 μL of samples were injected at 25 splitless time into a multi-baffled glass liner with injection temperature of 50 °C ramped to 250 °C by 12 °C s^−1^. Oven temperature was programmed at 50 °C for 1 min, ramp at 20 °C per minute to 330 °C which was held constant for 5 min. Data processing and data reporting were done by NIH Centre for Metabolomics.

Raw results data were normalized to reduce the impact between-series drifts of instrument sensitivity, caused by machine maintenance, aging, and tuning parameters. We used a variant of vector normalization in which the sum of all peak heights for all identified metabolites excluding the unknown for each sample was calculated and termed as mTIC. mTIC was used to avoid the potential non-biological artifacts for the biological normalizations, such as column bleed, plasticizers, or other contaminants. mTIC averages were determined between different treatment groups and following equation was used for normalization of metabolite i of sample j:

$$ {\mathrm{Metabolite}}_{\mathrm{ij},\ \mathrm{normalized}} = \left({\mathrm{metabolite}}_{\mathrm{ij},\ \mathrm{raw}}/\ {\mathrm{mTIC}}_{\mathrm{j}}\right) \times {\mathrm{mTIC}}_{\mathrm{average}} $$

This normalization is relative semi-quantification and expressed as normalized peak heights.

### Quantitative metabolite set enrichment analysis

MSEA is a way to identify biologically meaningful patterns that are significantly enriched in quantitative metabolomics data and was carried out using the tool from MetaboAnalyst [28, 52]. Over-representation analysis was implemented using the hypergeometric test to evaluate whether a particular metabolite set is represented more than expected by chance within the given compound list. One-tailed *P* values are provided after adjusting for multiple testing.

### Glucose-6 phosphatase assay

Fifty milligrams of liver/colon tissue were homogenized in 500 μL of RIPA buffer with protease inhibitors and the final homogenate was collected. Amount of inorganic phosphorus (Pi) released was assayed using Taussky-Shorr method [53]. Briefly, 150 μL of 100 mM Tris buffer (pH: 6.5) was mixed with 100 μL of 200 mM glucose-6 phosphate (Sigma-Aldrich Inc., USA; G7879) and incubated at 37 °C for 5 min. Subsequently, 10 μL of tissue homogenate was added, mixed, and incubated again at 37 °C for 5 min. The reaction was terminated by the addition of 90 μL of 10% TCA and incubation at 25 °C for 5 min. Finally, the mixture was centrifuged at 4000 rpm for 10 min and the supernatant was collected. The amount of Pi released was measured by mixing the supernatant or inorganic Pi solution (Sigma-Aldrich Inc., USA; P3869) with equal volume of Taussky-Shorr color reagent (10% ammonium molybdate prepared in 5 M sulphuric acid 10 mL, ferrous sulfate heptahydrate 5 g in 100 mL of distilled water) and incubated at 25 °C for 6 min. Finally, the absorbance was read at 660 nm. Specific glucose-6 phosphatase (G6Pase) activity was cleared of the contribution of non-specific phosphohydrolase activities by subtracting the activity toward 20 mMβ-glycerophosphate [54] (Sigma-Aldrich Inc., USA; G9422) and finally net G6Pase activity was expressed as μg of Pi released per mg of protein.

### Glycogen assay

A total of 100 mg of liver tissue was homogenized in 500 μL of 3% TCA and the homogenate was centrifuged at 3000 rpm for 5 min. Five volumes of cold 95% ethanol were added to the supernatant and left overnight at room temperature to precipitate glycogen. After a short spin for 10 s, the ethanolic supernatant was discarded and the pellet was dissolved in 250 μL of deionized water. Blank and standards (0.5 mg/mL of glucose) were prepared with same volume of deionized water. A total of 1.25 mL of anthrone reagent (anthrone 50 mg, thiourea 1 g, H_2_SO_4_ 72 mL in 100 mL deionized water) was added to all tubes and incubated at boiling temperature for 15 min. After cooling, the absorbance was measured at 620 nm against the blank. Amount of glycogen (mg/100 g of tissue) = DU/DS × 0.1 × volume of extract/gram of tissue × 100 × 0.9 where DU = absorbance of samples and DS = absorbance of glucose standard [55].

### Administration of sodium acetate

The mice were fasted for 4 h prior to the experiment. NaAc (100 mg/ kg body weight) was administered either orally using gavage or by RI continuously for seven days. Before RIs, the mice were handled gently and allowed to defecate and the complete defecation was confirmed by softly pressing at the distal end of the rectum. The mice were handled inversely and NaAc was administered in a maximum volume of 20 μL using 2–20 μL tips *via* micropipette. Finally, OGTT was performed by standard protocols. The animals were sacrificed a minimum of 24 h after OGTT and the organs were harvested.

### Collection of human fecal samples

Fecal samples were collected from the diabetic (n = 60) and control people (n = 60) from the population earlier studied for HbA_1c_ and OP analysis. People with bowel or stomach-related issues were excluded from the study. The control volunteers were confirmed for absence of obesity, hypertension, dyslipidemia, or other issues. The collection protocol was approved by the internal research and review board, ethical clearance, biosafety, and animal welfare committee of Madurai Kamaraj University. In addition, the project details were explained and their details in previous questionnaire were reconfirmed and new informed consent was obtained (Additional file 6). Subsequently, the next day early morning fecal samples were collected and stored immediately in ice. The samples were transported to the laboratory within 1 h and stored in −80 °C. The analysts performing the fecal parameters were blinded and unaware of the diabetic or OP exposure status of the samples.

### Short chain fatty acid quantification in feces

A total of 100 mg of feces was weighed and suspended in 2 mL of sterile PBS and vortexed for 1 min. The mixture was centrifuged at 3000 × g for 10 min. Five microliters of the supernatant was diluted 1:100 with sterile PBS. Five microliters of ethyl butyrate (Sigma-Aldrich; 109959) was added as internal standard to a final concentration of 5 mM. Subsequently, 250 μL of concentrated HCl was added followed by the addition of 1 mL of diethyl ether (Merck; LiChrosolv). The mixture was vortexed for 1 min and centrifuged at 3000 × g for 10 min. A total of 750 μL of upper phase was collected and derivatized with 120 μL of *N*-*tert*-Butyldimethylsilyl-*N*-methyltrifluoroacetamide (MTBSTFA) containing 1% tert-butyldimethylchlorosilane (TBDMSCI) (Sigma-Aldrich Inc., USA; 375934) by incubating at 80 °C for 20 min. The mixture was incubated at room temperature for 48 h to ensure complete derivatization. Gas chromatography was executed as described by Frost et al. [56] by flame ionization detector. The GC injector and detector temperatures were set at 275 °C. The oven temperature program was optimized to hold at 63 °C for 3 min and then to increase by 10 °C min^−1^ up to 190 °C. Helium gas was used as carrier gas. The transfer line temperature was adjusted to 280 °C. External standards for acetate were prepared at concentrations of 25, 12.5, 6.25, 1.25, and 0.625 mM and ethyl butyric acid was used as the internal standard at a concentration of 100 mM. Reported values were normalized according to the weight of original sample used.

### Statistics

All statistical analyses were performed using the statistical softwares SPSS version 20.0 and GraphPad Prism version 6.01. For association studies in survey, age and sex adjusted ORs and 95% CIs were calculated. For human studies, the non-parametric Mann–Whitney U test was employed. Pearson correlation and linear regression were performed to demonstrate the strength of relationship between two parameters. Plasma OP residues were categorized in quartiles based on the weighted sample distribution. For each OP, we used logistic regression to estimate ORs and CI levels for diabetes by comparing each quartile with the lowest quartile. We included likely or suspected confounders in models based on previously published data. In each analysis, we also evaluated the significance of the differences of the average proportion of diabetics across the four quartiles of the model by a generalized maximum likelihood Wald χ2 test. Our regression models were fitted with appropriate degrees of adjustment. We adjusted for age, sex, familial diabetic history, and BMI.

The following statistical analyses were used for animal studies: a two-way ANOVA with Bonferroni post-hoc analysis was used to compare between groups in different time-points and one-way ANOVA with Tukey’s post-hoc analysis or unpaired two-sided Student t-test was used to compare either between multiple or between two groups, respectively. The batch difference between replicate/triplicates were studied by a two-way ANOVA with Bonferroni post-hoc analysis. In all relevant panels, symbols, bars, or horizontal lines represent the mean and error bars represent s.e.m. For mouse experiments, cohort sizes match common practice of the described experiments and are repeated twice or thrice. For human experiments, sample size was chosen to validate statistical analyses. No data points were excluded from analyses in mice or human studies. *P <* 0.05 was considered statistically significant in all analyses. *P < 0.05, **P < 0.01, ***P < 0.001, ****P < 0.0001.

## Additional files

Additional file 1: Supplementary information document with all supplementary figures and their legends (Figure S1–Figure S14). (PDF 7418 kb)
Additional file 2: Supplementary information document with all supplementary tables and their legends related to human experiments (Table S1–Table S9). (XLSX 199 kb)
Additional file 3: Questionnaire used for studying the association between OP exposure and self-reported diabetes. (PDF 43 kb)
Additional file 4: Supplementary information document with all supplementary tables and their legends related to animal experiments (Table S10–Table S28). (XLSX 106 kb)
Additional file 5: Questionnaire used for collection of blood samples. (PDF 552 kb)
Additional file 6: Questionnaire used for collection of fecal samples. (PDF 434 kb)

## Abbreviations

AChE: acetylcholine esterase
CHL: chlorpyrifos
G6Pase: glucose-6 phosphatase
GNG: gluconeogenesis
MAL: malathion
MCP: monocrotophos
MPAR: methyl parathion
MRL: maximum residue limit
NaAc: sodium acetate
OP: organophosphates
SCFA: short chain fatty acids
TMDI: theoretical maximum daily intake
