# Supplementary material for: Gut microbial degradation of organophosphate insecticides-induces glucose intolerance via gluconeogenesis
Source: Genome Biol. 2017 Jan 24;18:8. doi: 10.1186/s13059-016-1134-6 (PMC5260025; doi:10.1186/s13059-016-1134-6)
Supplement: Additional file 1: — Supplementary information document with all supplementary figures and their legends (Figure S1–Figure S14). (PDF 7418 kb) [file 13059_2016_1134_MOESM1_ESM.pdf]

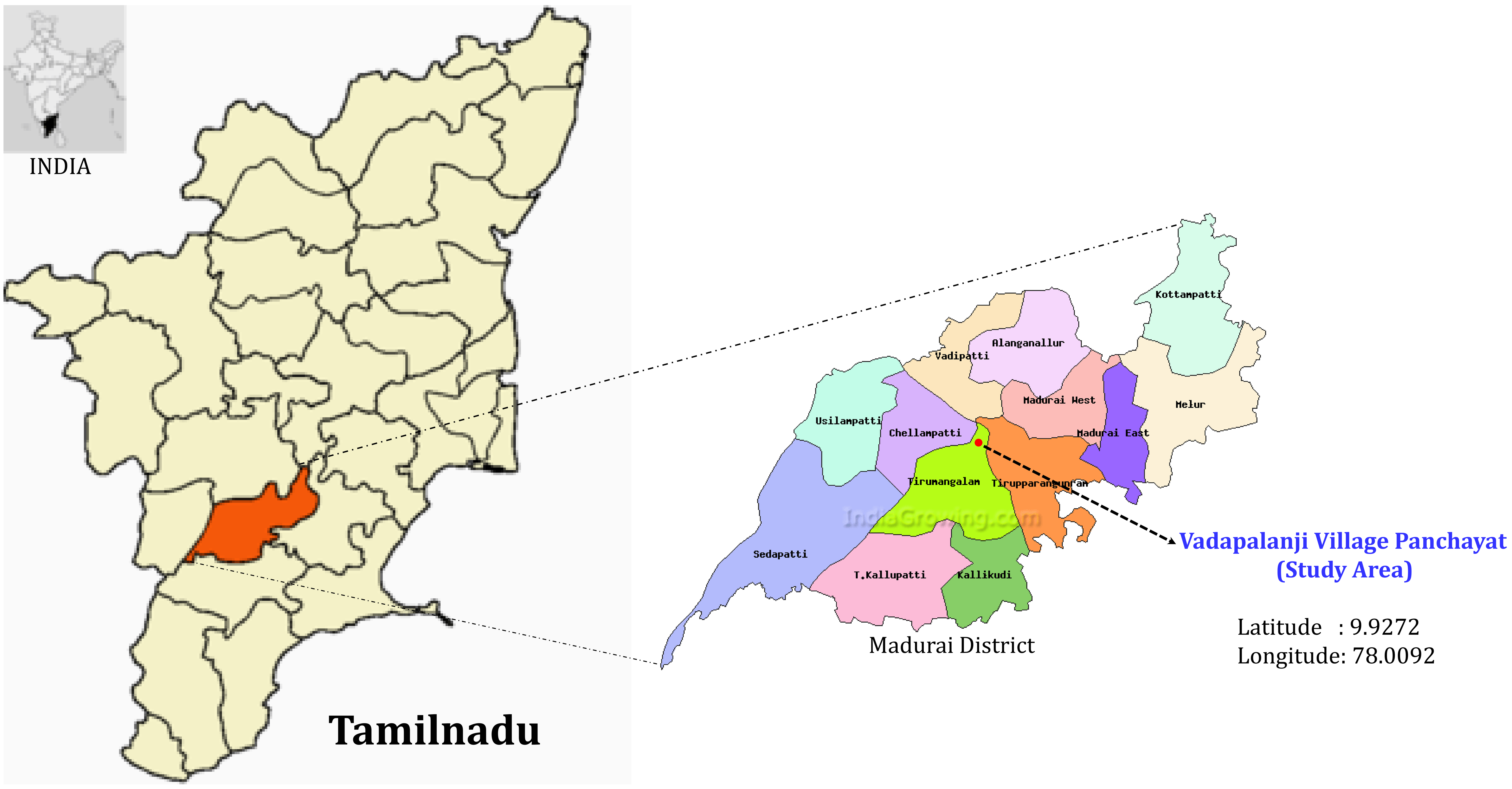

Figure S1 | Geographic location of the Study area. The study area chosen was Vadapalanji Village Panchayat located near Madurai Kamaraj University in Madurai District, Tamilnadu State of India.

a

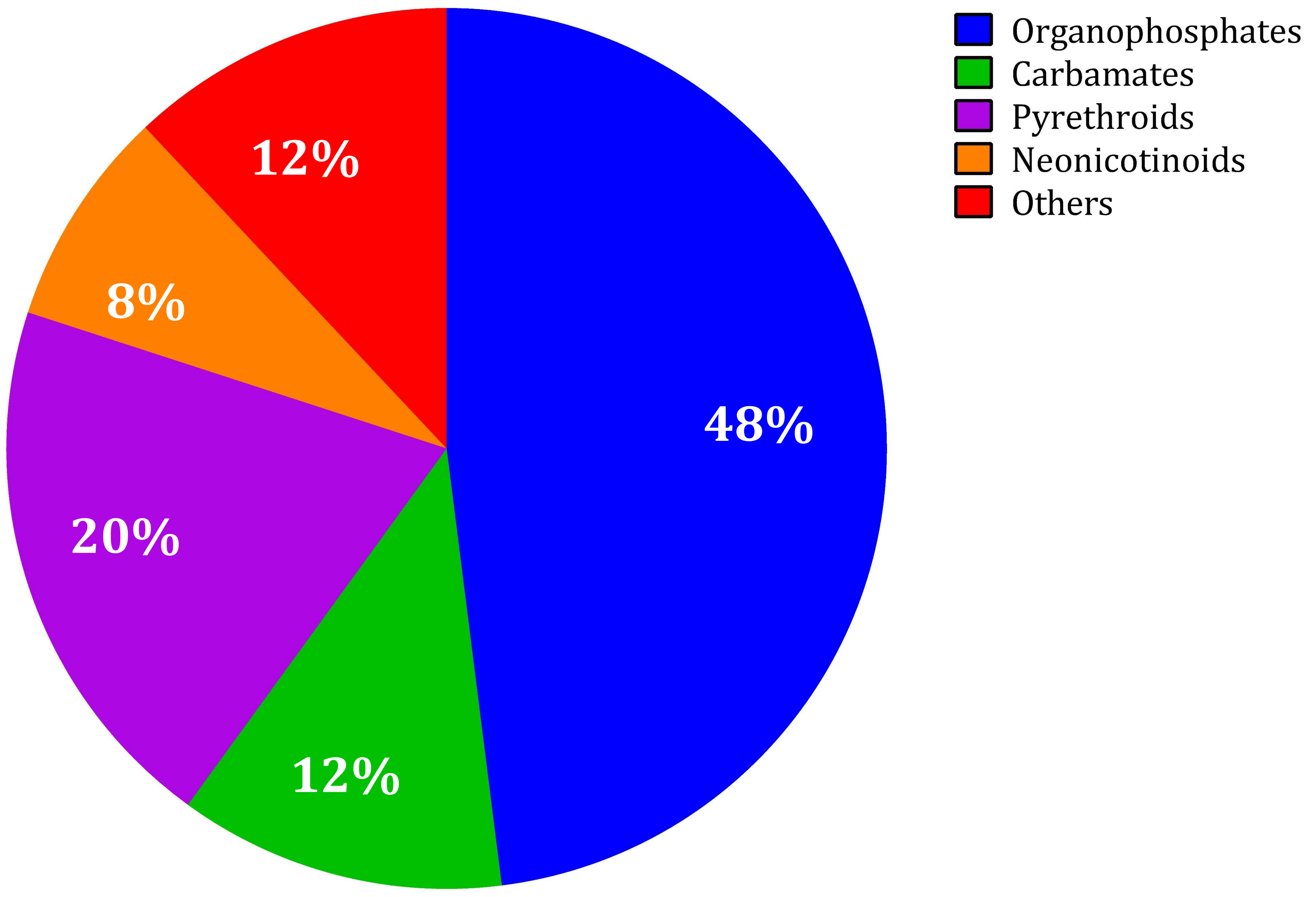

b

Total Number of Participants	3080
Number of participants directly exposed to OP	1686
Number of participants not directly exposed to OP	1394
Number of diabetic in exposed group	308
Number of diabetic in nonexposed group	86
Number of nondiabetic in exposed group	1378
Number of nondiabetic in nonexposed group	1308
**Odds ratio (age and sex adjusted)**	**1.3995**
95% Confidence interval	**0.738, 2.471**
z statistic	9.565<br>P<0.0001

c

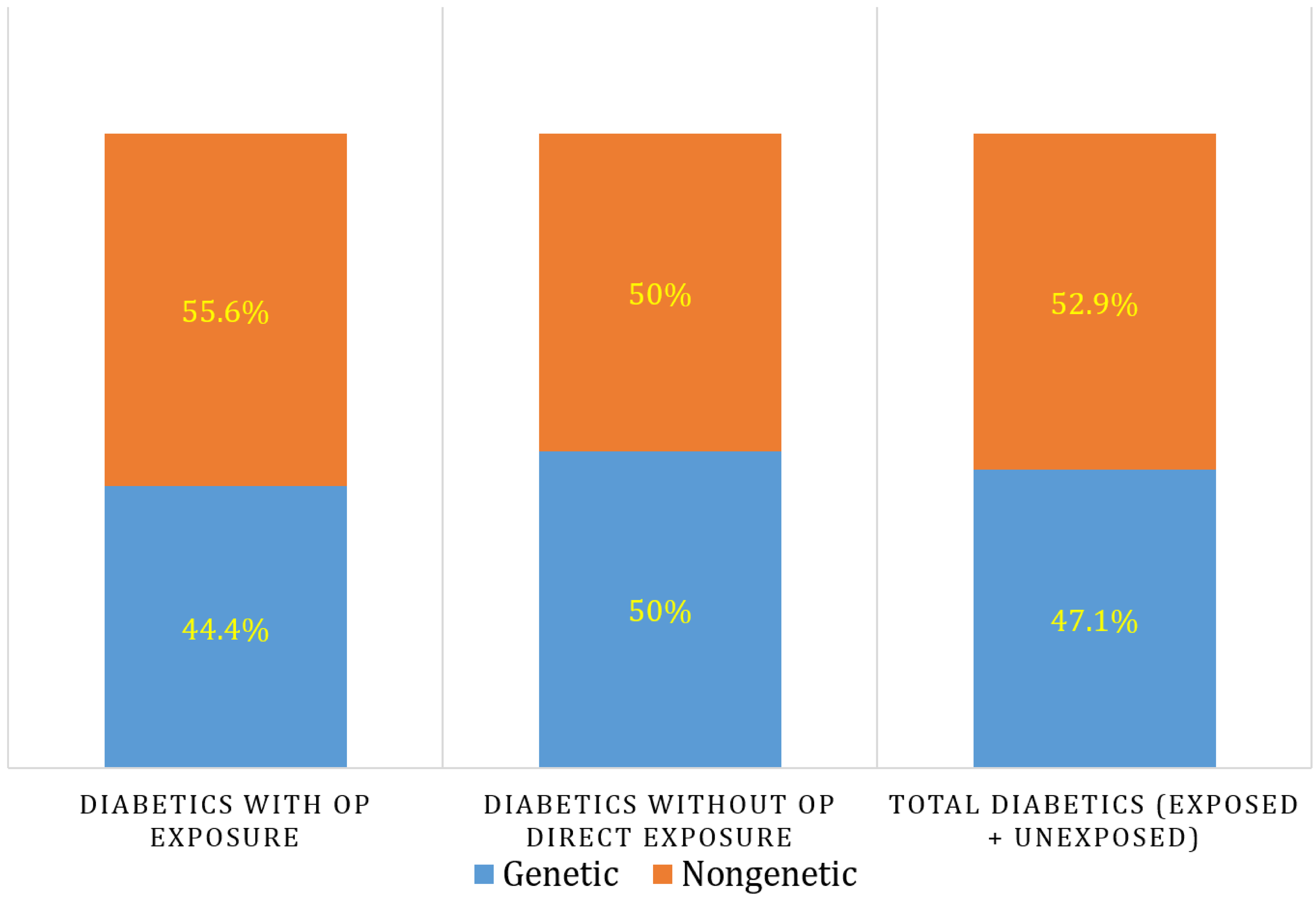

Figure S2| Organophosphate exposure associates with diabetic prevalence. Villagers around Madurai Kamaraj University were surveyed for exposure to organophosphates and diabetic prevalence. **a.** Frequency of usage of different types of insecticides in the study area. **b.** Characteristics of study population, odds ratio, 95% confidence interval and z statistic. **c.** Prevalence of genetic familial diabetic history among the diabetics with and without direct OP exposure (*N*=3080). The percentages of each group are mentioned within the slice of pie chart or bars.

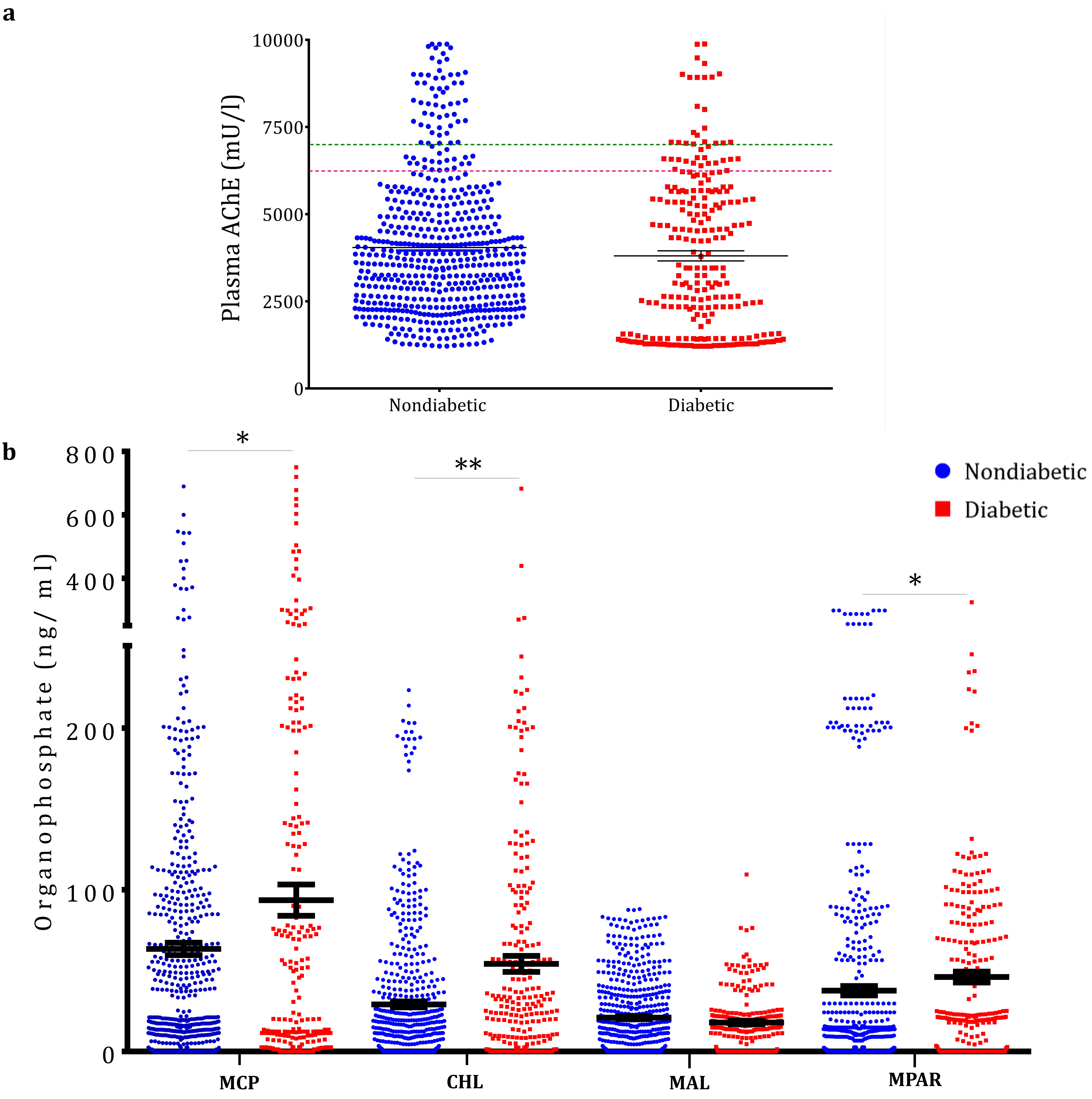

Figure S3| Validation of organophosphate exposure between diabetic (*N*=554) and nondiabetic (*N*=248) individuals **a.** Plasma acetylcholine esterase activity of diabetic and nondiabetic individuals. Horizontal dotted lines represents the reference values for males (green) and females (pink). **b.** Plasma MCP, CHL, MAL and MPAR residues. Horizontal lines represent mean; error bars represent s.e.m; **P<0.01, *P<0.05 Rank sum**,** Mann-Whitney U Test.

a

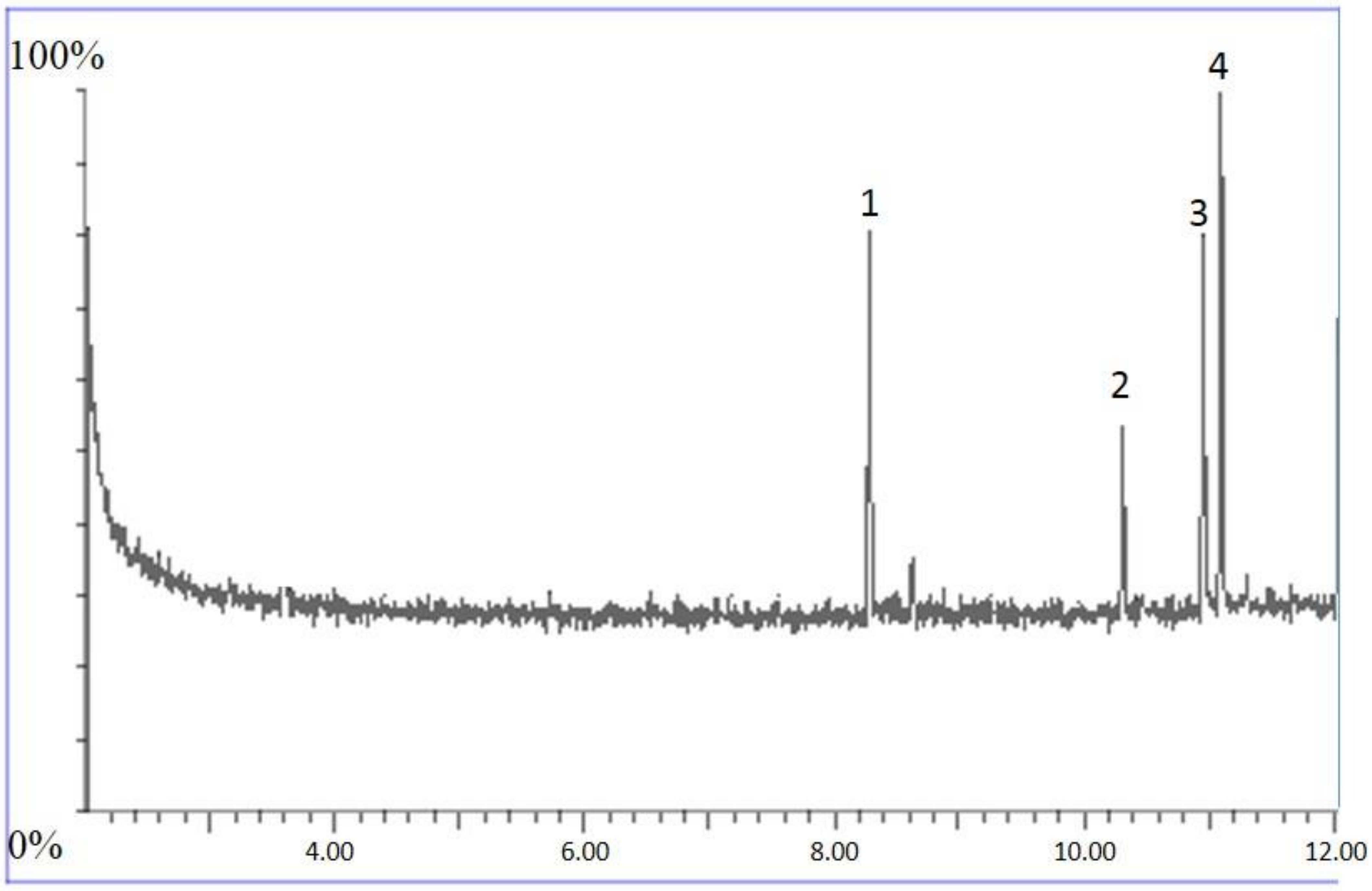

b

Peak No.	Insecticide	Retention Time (Minutes)	m/z fragments for SIM Mode
1	Monocrotophos	8.20	67,97,109,127,192
2	Methyl parathion	10.31	79,109,125,263
3	Malathion	10.96	93,127,173,285
4	Chlorpyrifos	11.11	97,197,286,314

Figure S4| Gas chromatography/ Mass spectrometry analysis of organophosphate residues. a. Gas chromatogram for the four OP standards. **b.** Retention time and mass fragments monitored for the detection of each OP under single ion monitoring mode (SIM).

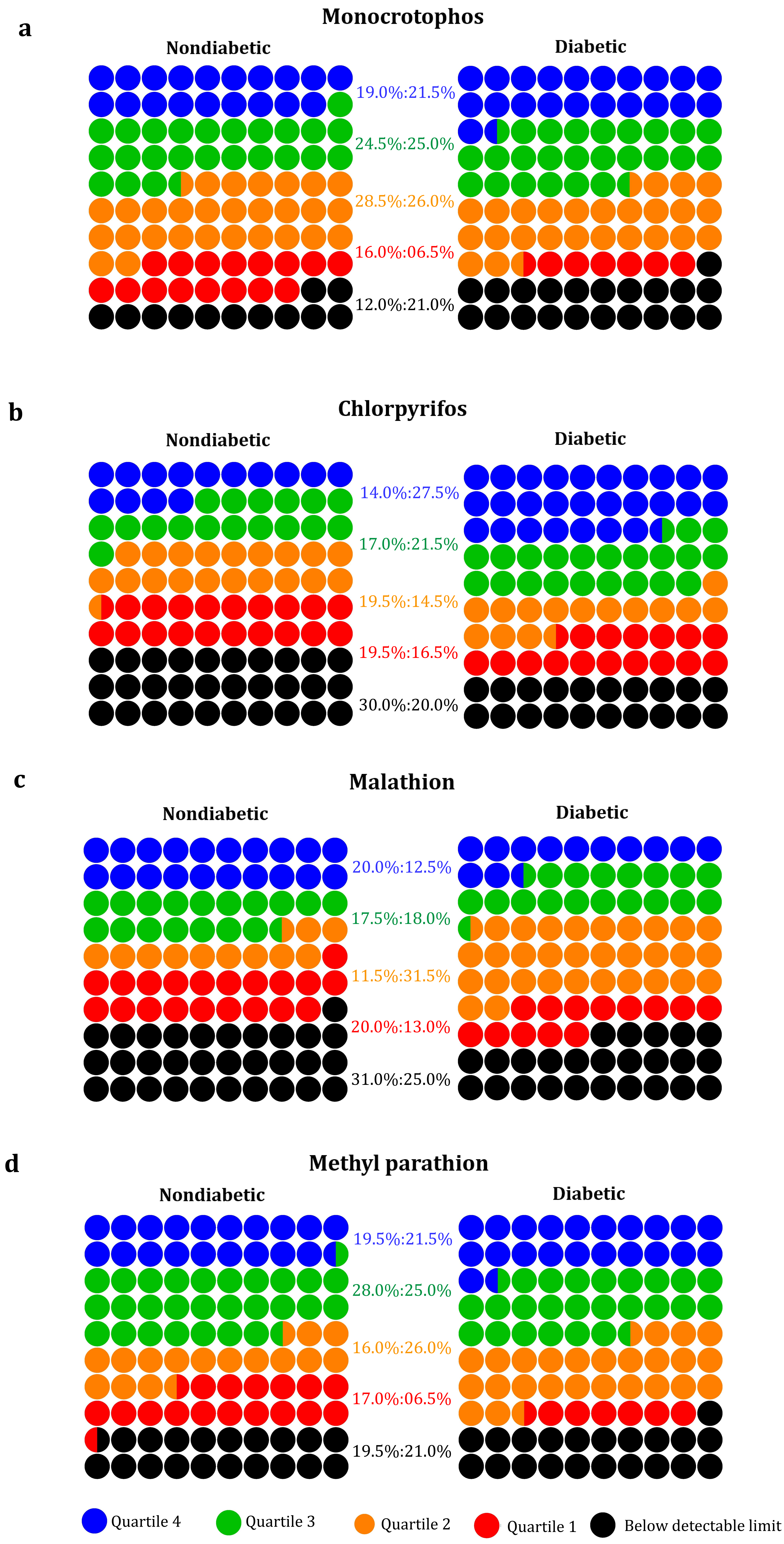

Figure S5. Interquartile analysis of plasma OP residues between nondiabetic and diabetic individuals. Nondiabetic (*N*=554) versus Diabetic (*N*=248) of **a.** MCP **b.** CHL **c.** MAL **d.** M.PAR. All the graphs provided represents a schematic diagram of percentage contribution of each factor. The squares has 10×10 circles and each circle represents one percent.

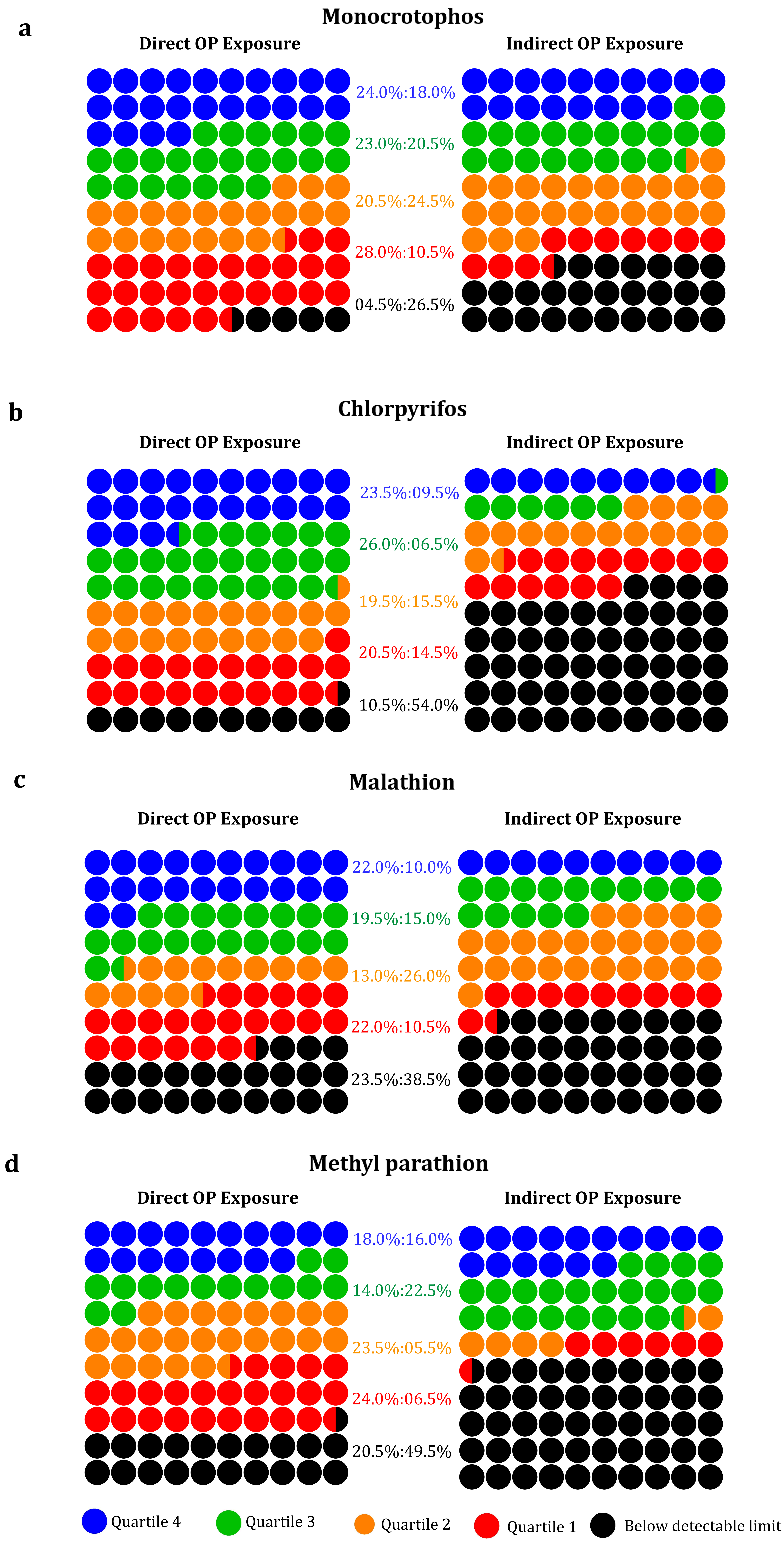

Figure S6. Interquartile analysis of plasma OP residues between individuals with direct and indirect OP exposure. Direct OP exposure (*N*=499) versus Indirect OP exposure (*N*=303) of **a.** MCP **b.** CHL **c.** MAL **d.** M.PAR. All the graphs provided represents a schematic diagram of percentage contribution of each factor. The squares has 10×10 circles and each circle represents one percent.

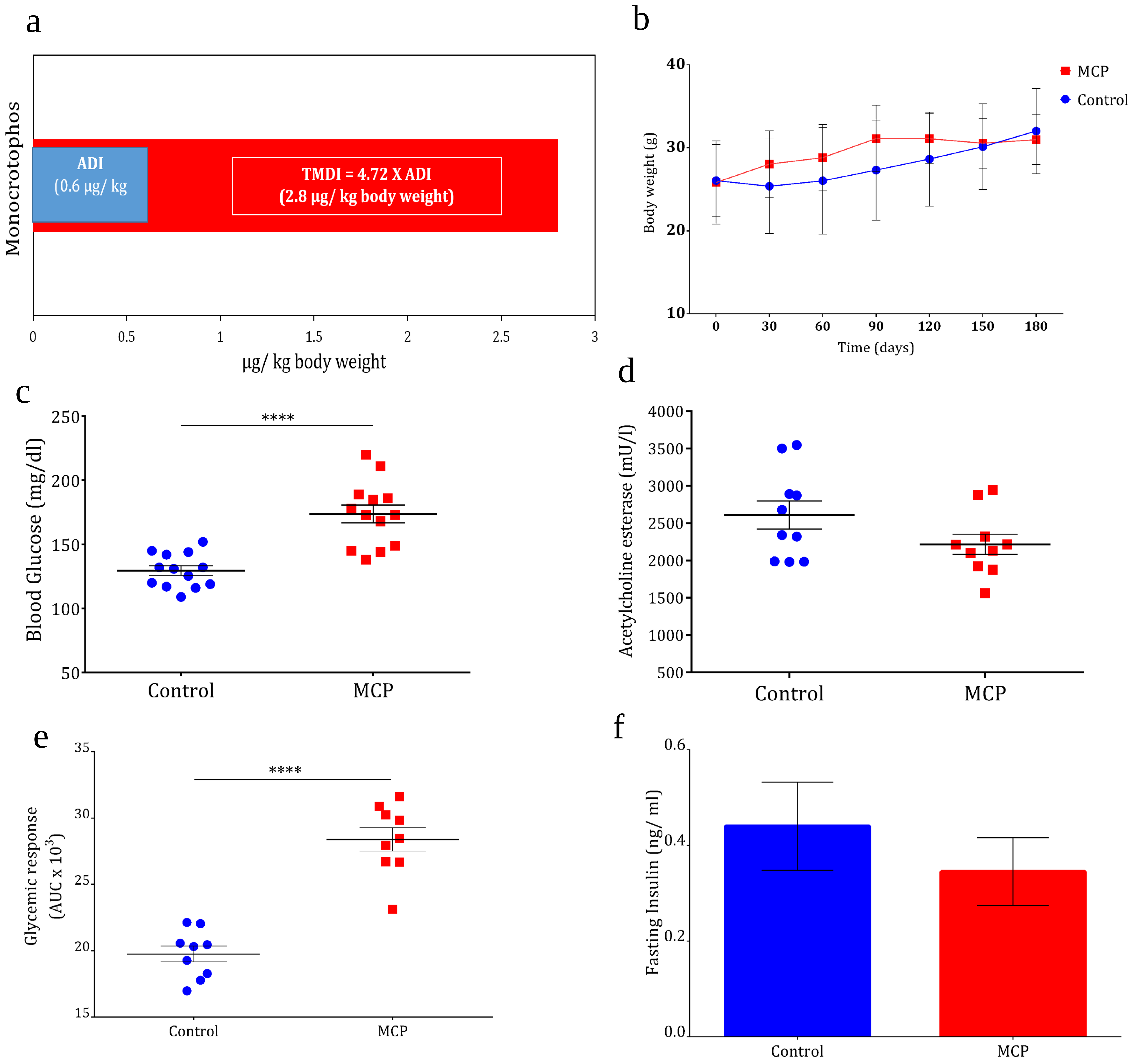

Figure S7 | Chronic organophosphate intake induces glucose intolerance. 8-week old *Balb/c* mice were treated with monocrotophos (MCP) at 10X TMDI dose for 180 days. **a.** Graph showcasing the fold difference between acceptable daily intake (ADI) and theoretical maximum daily intake (TMDI) for MCP. **b.** Periodical body weight control and MCP fed animals (*N*=13). **b.** Fasting blood glucose level of control and MCP group of animals measured after 180 days of treatment (*N*=13). **c.** Acetylcholine esterase (AChE) level of control and MCP fed animals (*N*=13). **d.** Oral glucose tolerance test represented as Glycemic response (AUC) of the mice drinking pure water and MCP mixed water (*N*=09). **e.** Fasting serum insulin levels of mice drinking pure water and MCP mixed water (*N*=04). Symbols (body weight & insulin) or horizontal lines represent means; error bars represent s.e.m. ****P<0.0001, Two-way ANOVA with Bonferroni correction (b) or unpaired two-sided Student *t*-test (c,d,e,f). Experiments were repeated thrice (b,c,d) or twice (e,f).

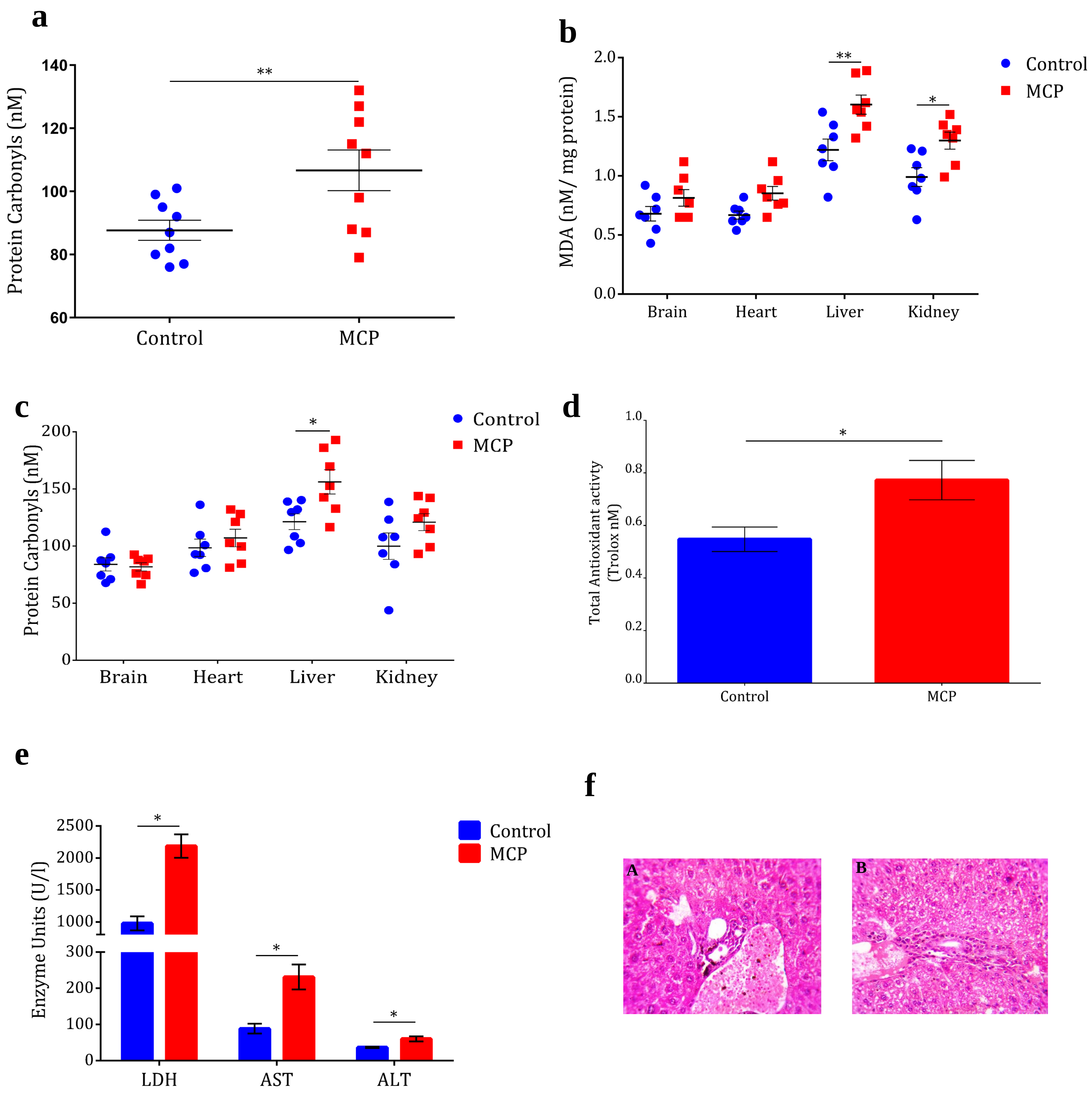

Figure S8 | OP-induced glucose intolerance induces oxidative stress and hepatic damage. 8-week old *Balb/c* mice were treated with monocrotophos (MCP) at 10X TMDI dose for 180 days. **a.** Protein carbonylation in serum of animals drinking pure water or MCP mixed water (*N*=9) **b.** Lipid peroxidation level in the major organs of control and MCP treated animals (*N*=07). **c.** Level of protein carbonyls in the major organs of control and MCP treated animals (*N*=07). **d.** Total antioxidant activity in the serum of control and MCP fed animals (*N*=04). **e.** Serum levels of hepatic damage markers (LDH, AST & ALT) of control and MCP group of animals measured after 180 days of treatment (*N*=04). **f.** Histopathology (400X) of the liver tissue of control (A) and MCP treated animals (B). Symbols, bars or horizontal lines represent means; error bars represent s.e.m. *P<0.05, **P<0.01. Unpaired two-sided Student *t*-test. Experiments were repeated twice.

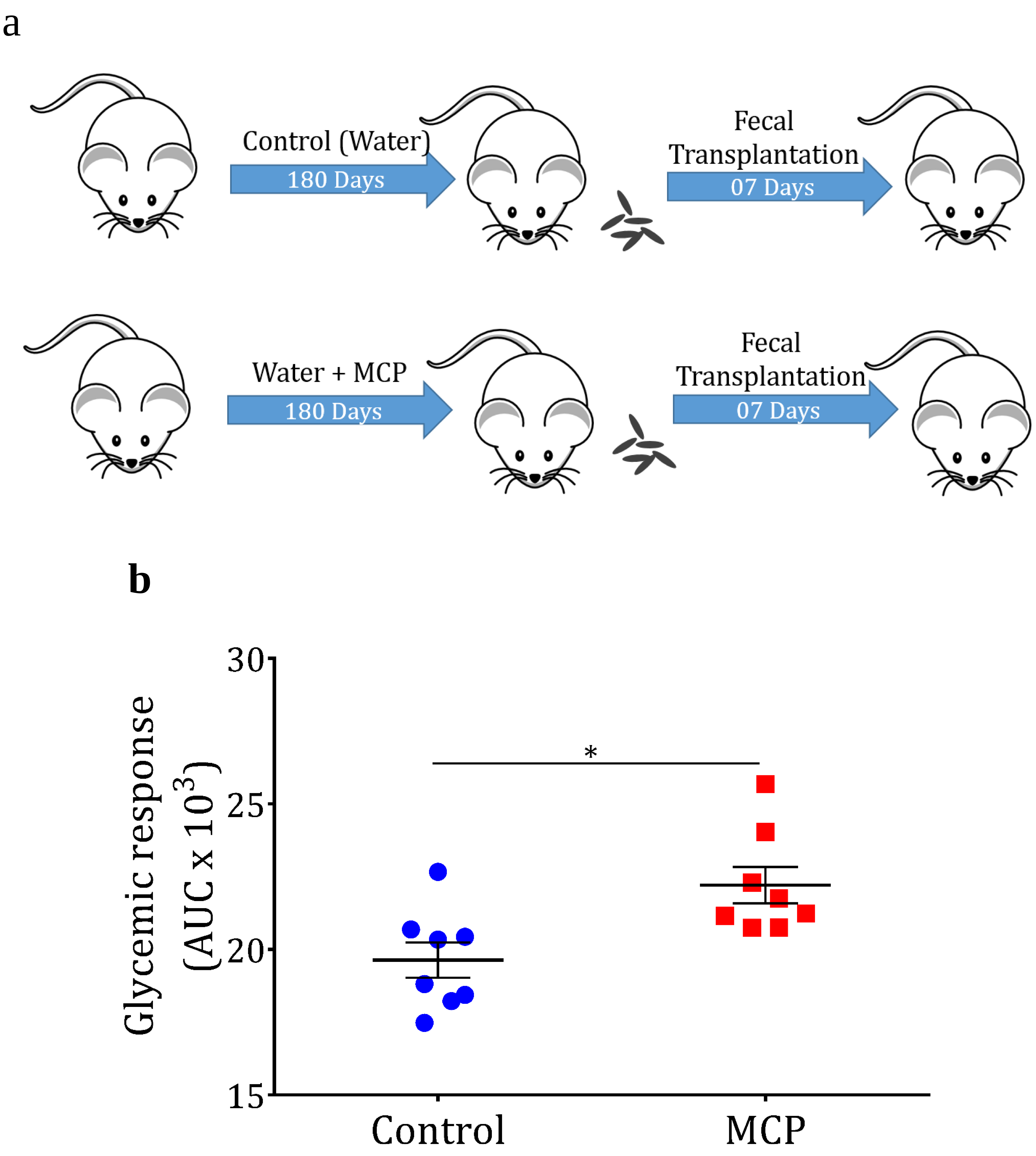

Figure S9 | Gut microbiota mediates OP-induced glucose intolerance. **a.** Schematic representation of faecal transplantation experiment. Faecal samples were collected. from control and MCP fed animals for 180 days and transplanted to randomly selected animals for seven days. **b.** Glycemic response of the animals transplanted with faeces from control and MCP treated animals (*N*=8). Horizontal lines represent means; error bars represent s.e.m. *P <0.05, Unpaired two-sided student *t*-test. Experiments were repeated twice.

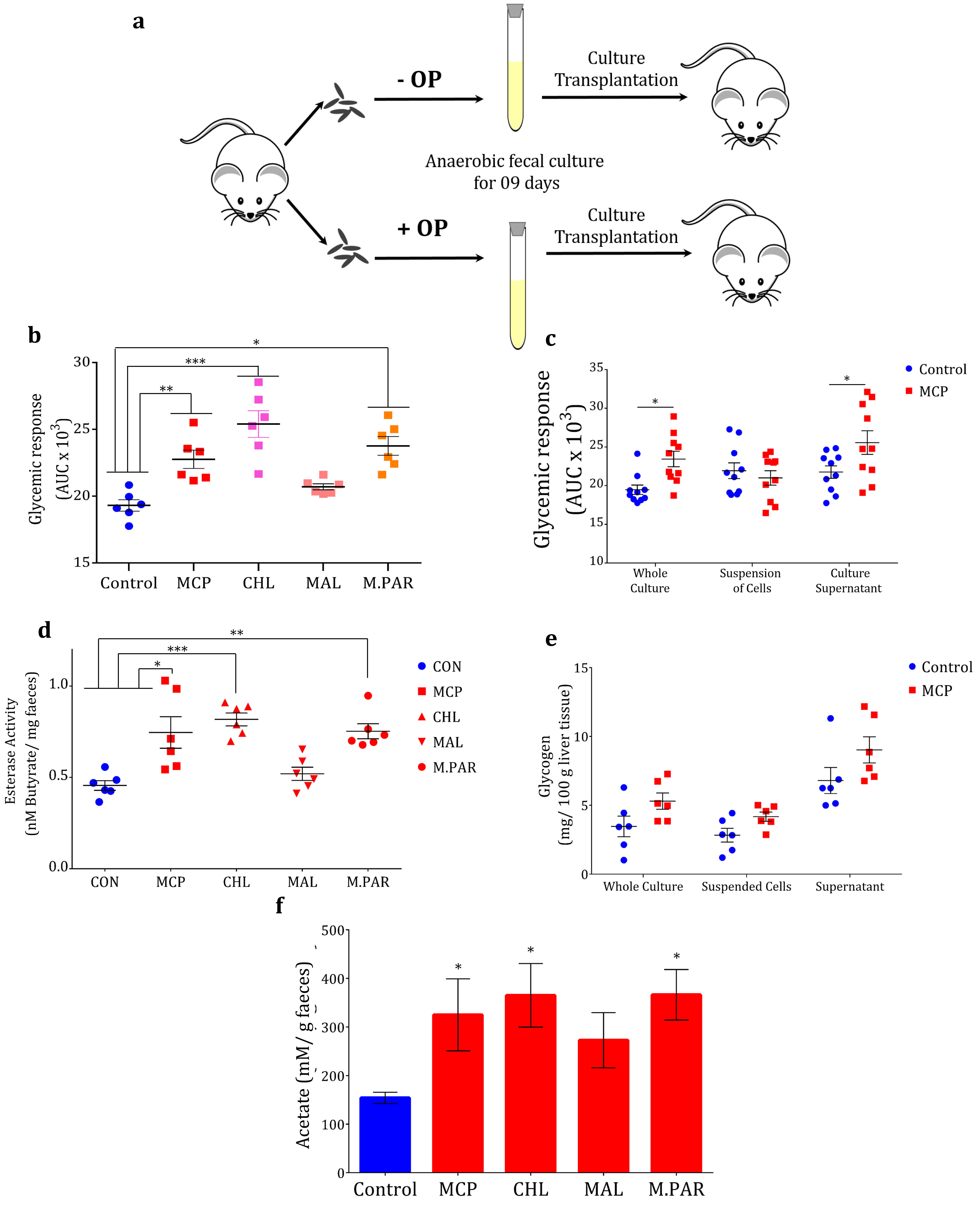

Figure S10 | Microbial degradation of OP-induces glucose intolerance. 8-week old Balb/c mice were treated with faecal cultures grown in presence or absence of OP. **a.** Schematic representation of culture transplantation experiment. **b.** Oral glucose tolerance test represented as glycemic response (AUC × 10^3) of the animals fed with faecal cultures (N=6). **c.** Glycemic response of the animals fed with whole culture, suspended cells or supernatant of MCP culture (N=10). **d.** faecal esterase activity of the animals fed with faecal cultures of different OP (N=06). **e.** Liver glycogen content animals fed with whole culture, suspended cells or supernatant of MCP culture (N=06). **f.** Faecal acetate level of the animals fed with faecal cultures of different OP (N=03). Horizontal lines or bars represent means; error bars represent s.e.m. ****P<0.0001, ***P<0.001, **P<0.01, *P<0.05. One-way ANOVA with Tukey post-hoc analysis (b,d,f) or unpaired two-sided Student t-test (c,e). Experiments were repeated twice.

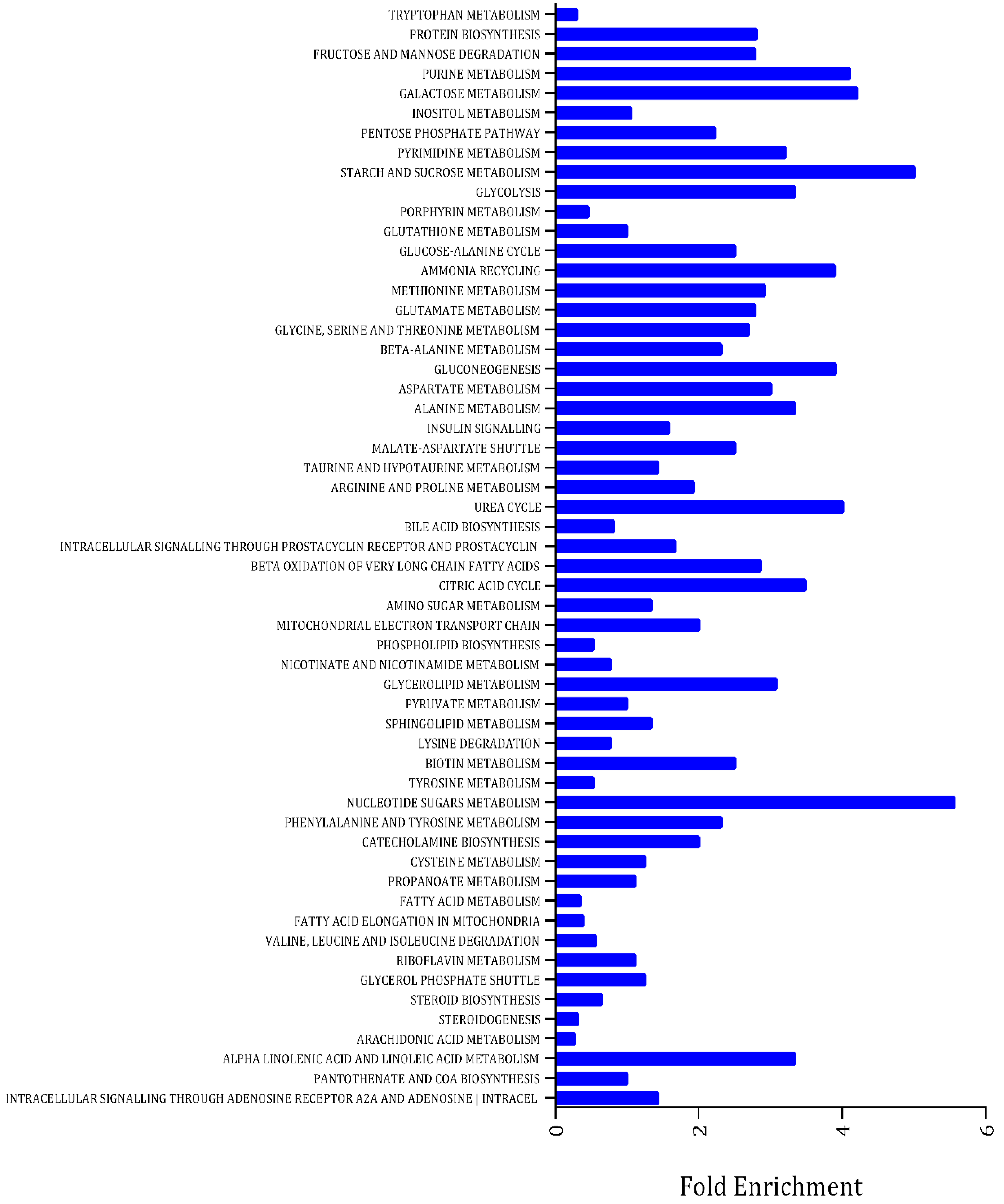

Figure S11| Quantitative Metabolite Set Enrichment Analysis. Enriched pathways analyzed by MetaboAnalyst based on the quantitative metabolite set enrichment analysis of whole metabolite profiling of cecum tissues (*N*=03).

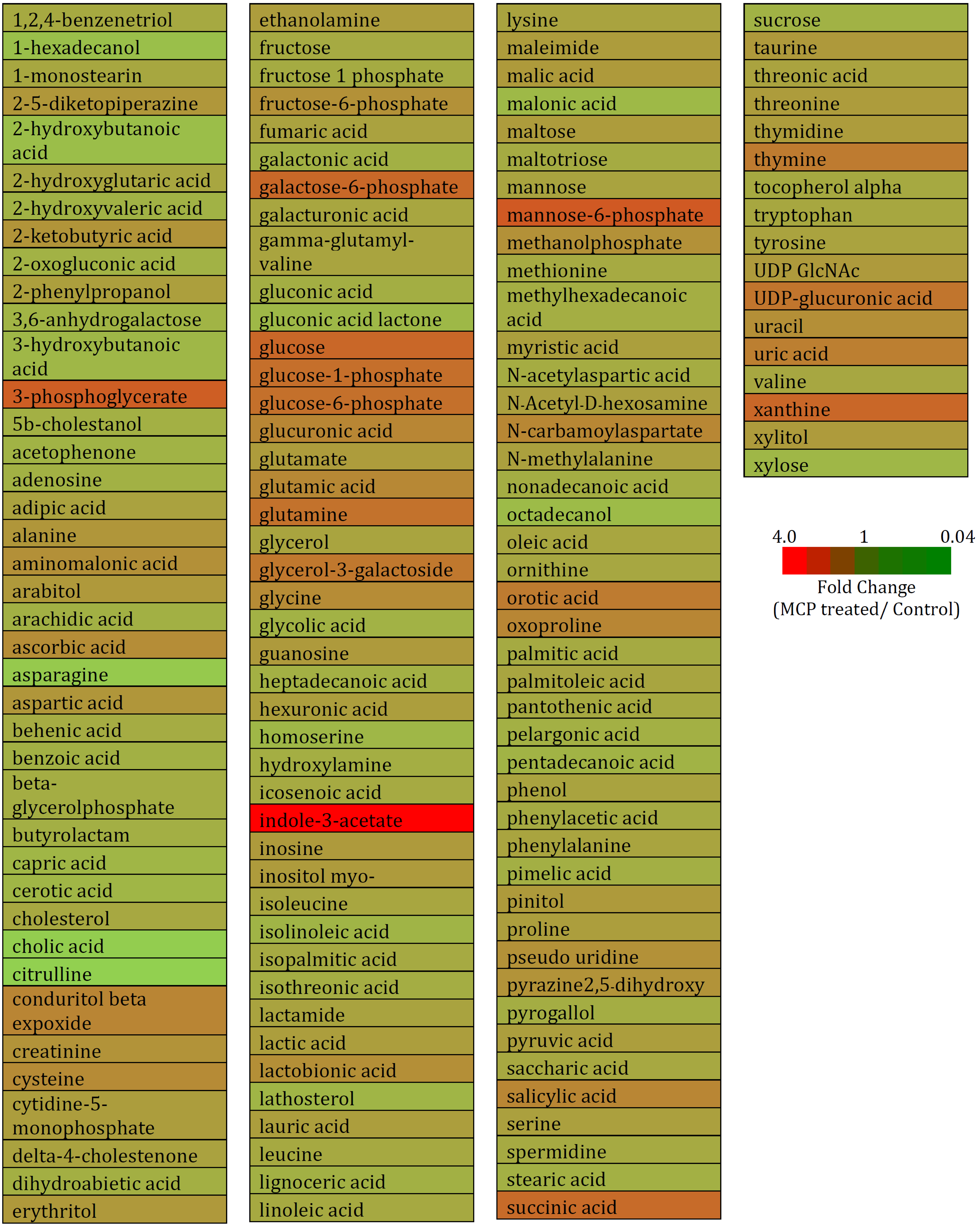

Figure S12 | Differential expression of host metabolites. Heat map elucidating the differential expression in fold change of host metabolites in caecum tissue on chronic exposure to OP (*N*=03).

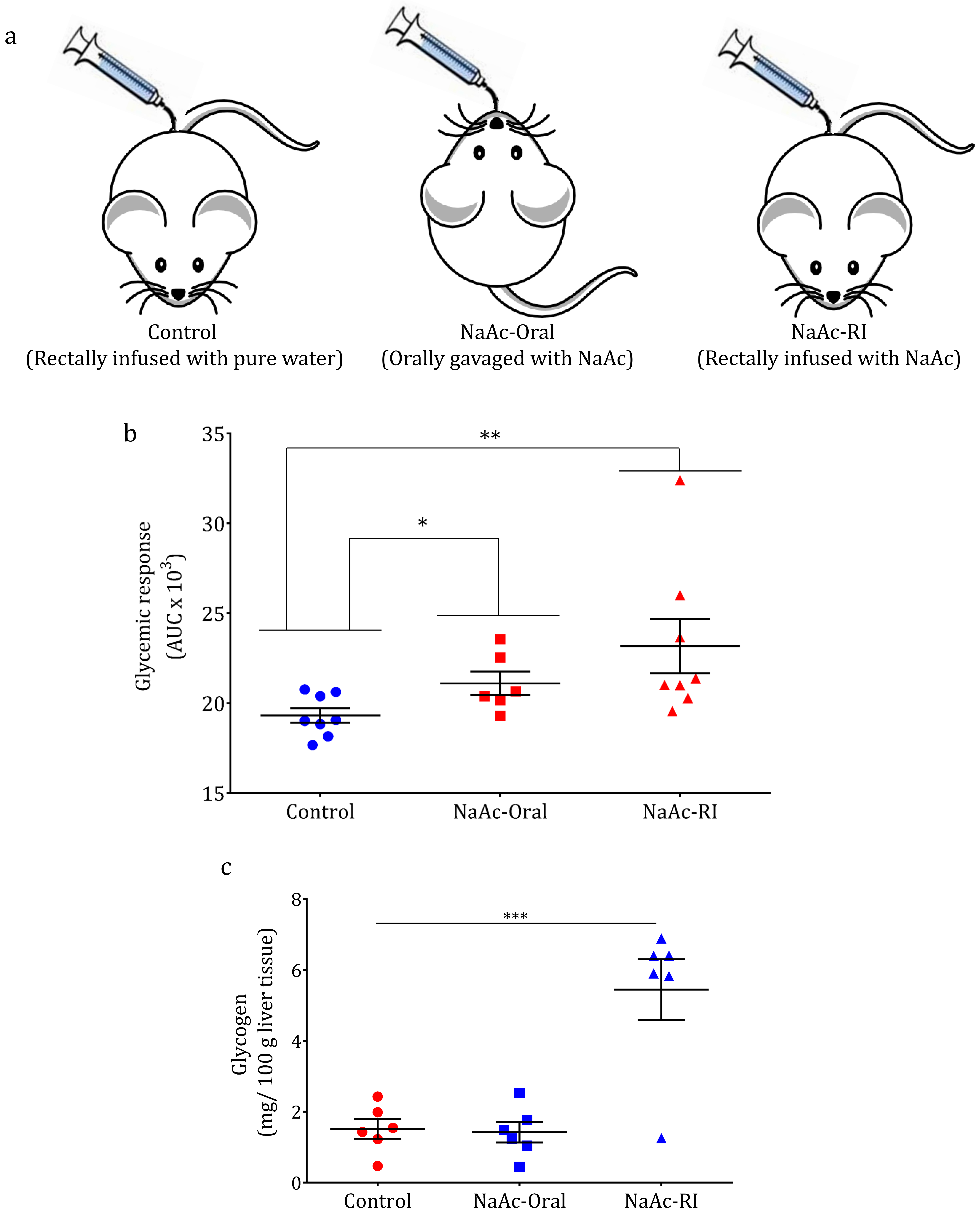

Figure S13 | Rectal infusion of sodium acetate induces glucose intolerance. a. Schematic illustration of oral and rectal infusion of sodium acetate treatment in mice. **b.** Oral glucose tolerance test represented as glycemic response (AUC × 10^3) of animals treated orally and by rectal infusion with sodium acetate (N=08). **c.** Liver glycogen content of the mice treated orally and by rectal infusion with sodium acetate (N=06). Horizontal lines represent means; error bars represent s.e.m. ***P<0.001, **P<0.01, *P<0.05. One-way ANOVA with Tukey post-hoc analysis. Experiments were repeated twice.

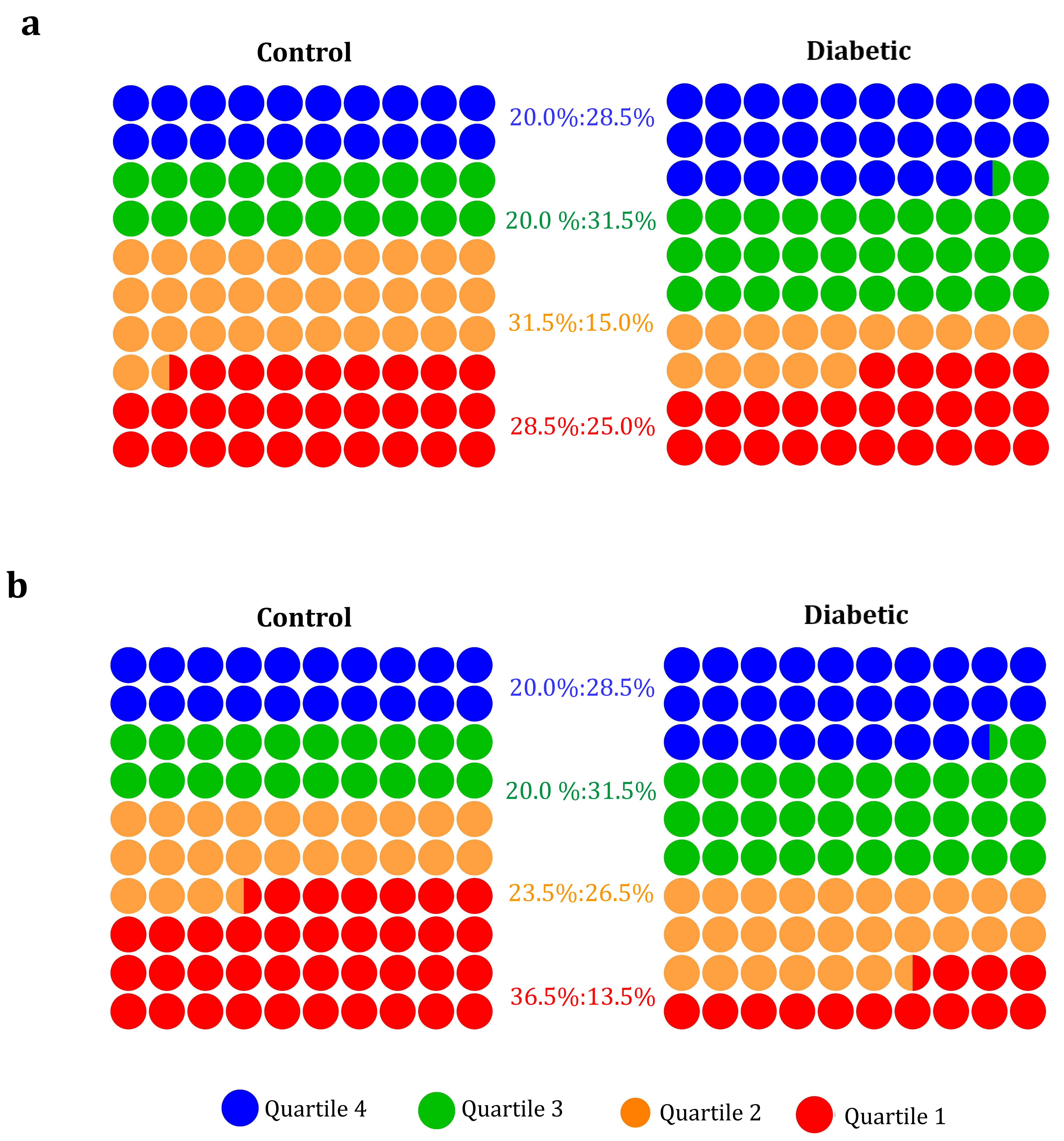

Figure S14. Interquartile analysis of faecal parameters between diabetic and diabetic individuals. Control (*N*=60) versus Diabetic (*N*=60) of **a.** Faecal esterase activity **b.** Faecal acetate content. All the graphs provided represents a schematic diagram of percentage contribution of each factor. The squares has 10×10 circles and each circle represents one percent.
