# Supplementary material for: Gut microbial degradation of organophosphate insecticides-induces glucose intolerance via gluconeogenesis
Source: Genome Biol. 2017 Jan 24;18:8. doi: 10.1186/s13059-016-1134-6 (PMC5260025; doi:10.1186/s13059-016-1134-6)
Supplement: Additional file 3: — Questionnaire used for studying the association between OP exposure and self-reported diabetes. (PDF 43 kb) [file 13059_2016_1134_MOESM3_ESM.pdf]

Survey on Association between Diabetic Status and Direct Exposure to Organophosphates

S. No.	Name	Sex	Age	Diabetic Status		Direct Exposure to Organophosphates	
				Yes	No	Yes	No
				Yes	No	Yes	No
				Yes	No	Yes	No
				Yes	No	Yes	No
				Yes	No	Yes	No
				Yes	No	Yes	No
				Yes	No	Yes	No
				Yes	No	Yes	No
				Yes	No	Yes	No
				Yes	No	Yes	No
				Yes	No	Yes	No
				Yes	No	Yes	No
				Yes	No	Yes	No
				Yes	No	Yes	No
				Yes	No	Yes	No
				Yes	No	Yes	No
				Yes	No	Yes	No
				Yes	No	Yes	No
				Yes	No	Yes	No
				Yes	No	Yes	No
				Yes	No	Yes	No
				Yes	No	Yes	No
				Yes	No	Yes	No
				Yes	No	Yes	No
				Yes	No	Yes	No
				Yes	No	Yes	No
				Yes	No	Yes	No
				Yes	No	Yes	No
				Yes	No	Yes	No
				Yes	No	Yes	No
