# Supplementary material for: Gut microbial degradation of organophosphate insecticides-induces glucose intolerance via gluconeogenesis
Source: Genome Biol. 2017 Jan 24;18:8. doi: 10.1186/s13059-016-1134-6 (PMC5260025; doi:10.1186/s13059-016-1134-6)
Supplement: Additional file 5: — Questionnaire used for collection of blood samples. (PDF 552 kb) [file 13059_2016_1134_MOESM5_ESM.pdf]

வரிசை எண்/ Serial No.:

வினாப்பட்டியல் 001/ Questionnaire 001

தேதி/ Date:

பெயர்/ Name			
பாலினம்/ Sex		வயது/Age	
எடை/ Weight		உயரம்/ Height	
கிராமம்/ Village			
தொழில்/ Occupation			
உணவு மற்றும் பிற பழக்கங்கள்/ Food and other Habits			
உணவு பழக்கங்கள்/ Food Habits	1. சைவம்/ Vegetarian<br>2. அசைவம் / Nonvegetarian		
மது<br>Alcohol Drinking	1. தினமும்/ Daily<br>2. எப்பொழுதாவது/ Occasionally	3. முன்பு/ Formerly<br>4. இல்லை/ Never	
புகை பிடித்தல்<br>Smoking	1. தினமும்/ Daily<br>2. எப்பொழுதாவது/ Occasionally	3. முன்பு/ Formerly<br>4. இல்லை/ Never	
புகையிலை<br>Tobacco Chewing	1. தினமும்/ Daily<br>2. எப்பொழுதாவது/ Occasionally	3. முன்பு/ Formerly<br>4. இல்லை/ Never	
பூச்சிக்கொல்லிகளுடன் தொடர்பு/ Exposure history to pesticides			
பூச்சிக்கொல்லிகளுடன் தொடர்பு/ Exposure history to pesticides	1. பூச்சிக்கொல்லி தெளிப்பவர்/ Pesticide Applicator<br>2. வயலில் வேலை செய்பவர்/ Field Worker<br>3. பூச்சிக்கொல்லி கடையில் வேலை செய்பவர்/ Worker in pesticide shop<br>4. உணவின் முலம்/ via food chain		
Types of Pesticides being used:			

வரிசை எண்/ Serial No.:

**நோய்கள்/ Disease status**	
நீரிழிவு நோய்/ **Diabetes**	1. ஆம்/ Yes<br>2. இல்லை/ No
எத்தனை வருடங்கள்/ No. of Years	
பரம்பரையில் பிறருக்கு நீரிழிவு நோய் உண்டா? Familial History of diabetes	1. ஆம்/ Yes<br>2. இல்லை/ No
ஆம் எனில், உறவு முறையை குறிக்கவும்.If yes, mention the relationship	
நீரிழிவு நோயிற்காக உட்கொள்ளும் மருந்துகள்/ Medications for Diabetes	
கடந்த ஒரு மாதத்தில் ஏதேனும் வயறு போக்கு, வயிறு வலி Any bowel related problems or stomach pains in last one month	1. ஆம்/ Yes<br>2. இல்லை/ No
பிற நோய்கள் மற்றும் உட்கொள்ளும் மருந்துகள்/ Other diseases and medications for them	

முகவரி/ Address for Communication:

தொடர்பு எண்/ **Contact No.:**

வரிசை எண்/ Serial No.:

ஒப்புதல் தீர்மானம்/ Informed Consent

நான் எனது இரத்தத்தை ஆய்வுக்காக அளிக்கிறேன். எதற்காக இந்த ஆய்வு என்பதை படித்தோ அல்லது ஆய்வாளர்களிடமிருந்து கேட்டோ அறிந்து கொண்டேன். எனது சந்தேகங்களுக்கு உரிய விளக்கம் அளிக்கப்பட்டது. எனது இரத்தத்தை மற்றும் மலத்தை எனது முழு ஒப்புதலுடன் அளிக்கிறேன். ஒரு முறை அளித்த பிறகு, ஆய்வாளர்களிடமிருந்து மீண்டும் திரும்ப பெற முடியாது என்பதை நான் அறிவேன். மேலும் எனது விவரங்களை அறிவியல் ஆய்விதழில் பதிப்பிக்க முழு சம்பதம் தெரிவிக்கிறேன். எனது பெயர் ஆய்விதழில் வெளியாகது என்பதும் ஆனால் பிற விவரங்கள் வெளியாகும் என்பதையும் நான் அறிவேன். எனக்கு ஆய்வு கட்டுரையை படிக்கும் வாய்ப்பு வழங்கப்பட்டது. இந்த ஒப்புதல் வடித்தில் கையெழுத்து போடுவது, எனது தனியுரிமையை நீக்காது என்பதையும் நான் அறிவேன்.

I agree to give my blood sample for research purpose. I understood the need of the study by either reading or by listening to them. All my doubts if any were clarified by them. I am giving the samples with my full consent. I am also aware that I can’t withdraw my sample from the repository once it is given. I give my consent for publication of my information in scientific journals. I understand that information will be published without my name but that full anonymity cannot be guaranteed. I have been offered the opportunity to read the manuscript. Signing this consent from does not remove my rights of privacy.

தேதி/ Date :

இடம்/ Place :

கையொப்பம்/Signature

சேகரிப்பவரின் கையொப்பம்/
Signature of the person collecting samples
