# Supplementary material for: Gut microbial degradation of organophosphate insecticides-induces glucose intolerance via gluconeogenesis
Source: Genome Biol. 2017 Jan 24;18:8. doi: 10.1186/s13059-016-1134-6 (PMC5260025; doi:10.1186/s13059-016-1134-6)
Supplement: Additional file 6: — Questionnaire used for collection of fecal samples. (PDF 434 kb) [file 13059_2016_1134_MOESM6_ESM.pdf]

<u>வினாப்பட்டியல் 002/ Questionnaire 002</u>

தேதி/ Date:

வரிசை எண்/ Serial No.:

**பெயர்/ Name :**			
**பாலினம்/ Sex :**		**வயது/Age :**	

ஒப்புதல் தீர்மானம்/ Informed Consent

நான் எனது மலத்தை ஆய்வுக்காக அளிக்கிறேன். எதற்காக இந்த ஆய்வு என்பதை படித்தோ அல்லது ஆய்வாளர்களிடமிருந்து கேட்டோ அறிந்து கொண்டேன். எனது சந்தேகங்களுக்கு உரிய விளக்கம் அளிக்கப்பட்டது. எனது இரத்தத்தை மற்றும் மலத்தை எனது முழு ஒப்புதலுடன் அளிக்கிறேன். ஒரு முறை அளித்த பிறகு, ஆய்வாளர்களிடமிருந்து மீண்டும் திரும்ப பெற முடியாது என்பதை நான் அறிவேன். மேலும் எனது விவரங்களை அறிவியல் ஆய்விதழில் பதிப்பிக்க முழு சம்பதம் தெரிவிக்கிறேன். எனது பெயர் ஆய்விதழில் வெளியாகது என்பதும் ஆனால் பிற விவரங்கள் வெளியாகும் என்பதையும் நான் அறிவேன். எனக்கு ஆய்வு கட்டுரையை படிக்கும் வாய்ப்பு வழங்கப்பட்டது. இந்த ஒப்புதல் வடித்தில் கையெழுத்து போடுவது, எனது தனியுரிமையை நீக்காது என்பதையும் நான் அறிவேன்.

I agree to give my faecal samples for research purpose. I understood the need of the study by either reading or by listening to them. All my doubts if any were clarified by them. I am giving the samples with my full consent. I am also aware that I can’t withdraw my sample from the repository once it is given. I give my consent for publication of my information in scientific journals. I understand that information will be published without my name but that full anonymity cannot be guaranteed. I have been offered the opportunity to read the manuscript. Signing this consent from does not remove my rights of privacy.

தேதி/ Date :
இடம்/ Place :

கையொப்பம்/Signature

சேகரிப்பவரின் கையொப்பம்/
Signature of the person collecting samples
